# The Retrospective on Atypical *Brucella* Species Leads to Novel Definitions

**DOI:** 10.3390/microorganisms10040813

**Published:** 2022-04-14

**Authors:** Alessandra Occhialini, Dirk Hofreuter, Christoph-Martin Ufermann, Sascha Al Dahouk, Stephan Köhler

**Affiliations:** 1 Institut de Recherche en Infectiologie de Montpellier (IRIM), CNRS, University Montpellier, INSERM, 34293 Montpellier, France; alessandra.occhialini@irim.cnrs.fr; 2 German Federal Institute for Risk Assessment, 12277 Berlin, Germany; dirk.hofreuter@bfr.bund.de (D.H.); christoph-martin.ufermann@bfr.bund.de (C.-M.U.); sascha.al-dahouk@gmx.de (S.A.D.)

**Keywords:** *Brucella*, novel species, atypical species, classical species, core species, phylogeny, metabolism, acid stress, infection models

## Abstract

The genus *Brucella* currently comprises twelve species of facultative intracellular bacteria with variable zoonotic potential. Six of them have been considered as classical, causing brucellosis in terrestrial mammalian hosts, with two species originated from marine mammals. In the past fifteen years, field research as well as improved pathogen detection and typing have allowed the identification of four new species, namely *Brucella microti*, *Brucella inopinata*, *Brucella papionis*, *Brucella vulpis*, and of numerous strains, isolated from a wide range of hosts, including for the first time cold-blooded animals. While their genome sequences are still highly similar to those of classical strains, some of them are characterized by atypical phenotypes such as higher growth rate, increased resistance to acid stress, motility, and lethality in the murine infection model. In our review, we provide an overview of state-of-the-art knowledge about these novel *Brucella* sp., with emphasis on their phylogenetic positions in the genus, their metabolic characteristics, acid stress resistance mechanisms, and their behavior in well-established in cellulo and in vivo infection models. Comparison of phylogenetic classification and phenotypical properties between classical and novel *Brucella* species and strains finally lead us to propose a more adapted terminology, distinguishing between core and non-core, and typical versus atypical brucellae, respectively.

## 1. Introduction

Brucellosis is one of the major bacterial zoonotic diseases, usually indirectly transmitted from livestock to humans via the consumption of raw and unpasteurized animal food products. In endemic regions with a high prevalence of the disease in farm animals, direct transmission is also reported for professions in close contact with infected animals, such as veterinarians, farmers, and butchers. Laboratory-acquired infections are common especially in non-endemic regions, because of the low awareness of brucellosis and the low infectious dose by inhalation [1,2]. Human infections are most frequently caused by *Brucella melitensis* shed into sheep and goat milk with 500,000 cases annually notified worldwide [3]. While infected livestock suffers from a loss of productivity, reproductive failure, stillbirth, and abortion, human brucellosis is a systemic, febrile illness that may affect almost every organ system and can cause long-term sequelae.

**Figure 1:**
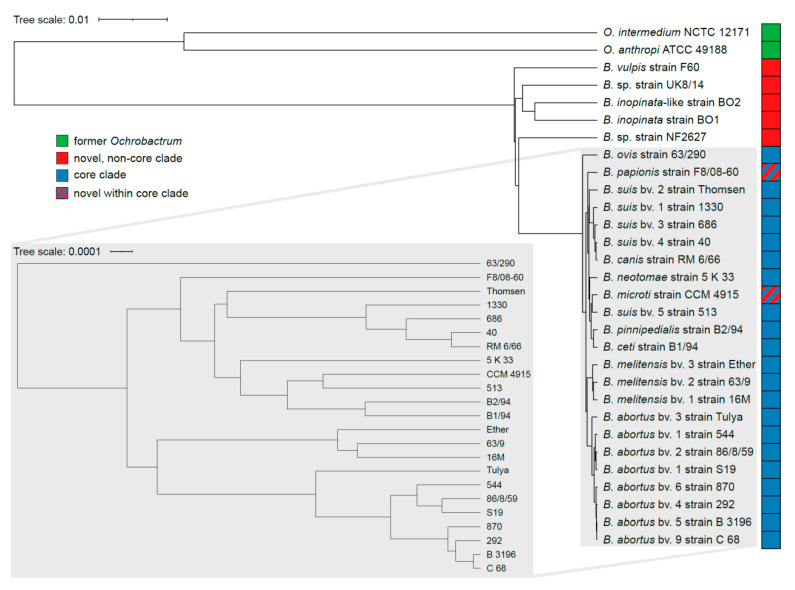
Phylogenetic tree of the genus *Brucella*. Genome assemblies of *Brucella* strains, including all core and novel type strains, as well as two former *Ochrobactrum* type strains, were used. Pairwise average nucleotide identities were calculated with fastANI (v1.32) [5]. Hierarchical clustering was conducted using the unweighted pair group with arithmetic mean (UPGMA) method. The phylogenetic tree was visualized with the EMBL online tool “Interactive Tree Of Life” (iTOL v6). The colored squares indicate affiliation to *Brucella* groups. The gray shaded area illustrates the phylogeny of the core clade in more detail.

For more than a century, only mammals have been considered as potential animal hosts of *Brucella* spp. The genus *Brucella* belongs to the family of Alphaproteobacteria and hitherto comprises twelve species, i.e., six classical and six novel species. Most recently, *Brucella* was merged with the phylogenetically closely related genus *Ochrobactrum* [4]. The classical *Brucella* species are a very homogeneous group of bacteria (average nucleotide identities (ANI) > 99%) with marked host preferences (Figure 1, [5]). *B. melitensis* (bv. 1–3) is mainly isolated from sheep, goats, and camels, *Brucella abortus* (bv. 1–7, 9) from cattle and buffaloes, *Brucella suis* depending on its biovar from pigs (bv. 1–3), hares and wild boars (bv. 2), reindeers (bv. 4), and rodents (bv. 5), *Brucella canis* is found in dogs, *Brucella ovis* in sheep, and *Brucella neotomae* in desert woodrats. The three species mentioned first are known as highly pathogenic for humans (except for *B. suis* bv. 2), while *B. canis* rarely infects humans [6] and only single cases have been reported for *B. neotomae* [7]. From marine mammals, two further species, *Brucella ceti* (whales, porpoises, and dolphins) and *Brucella pinnipedialis* (seals, sea lions, and walruses), have been isolated [8]. However, *B. ceti* sequence type ST27 is the only strain among the marine brucellae currently known to be pathogenic to humans [9]. Genomic sequence data allow for the description of various lineages in the marine subgroup and clearly separate isolates from dolphins from other *B. ceti*, suggesting a third marine species [10].

Historically, members of the genus *Brucella* were classified by their host preference, pathogenicity, and a few phenotypic characteristics including carbon dioxide (CO_2_) requirement, hydrogen sulfide (H_2_S) production, urease activity, dye sensitivity (basic fuchsin and thionin), lysis by specific bacteriophages, and agglutination with monospecific sera (anti-A/M) [11]. The classical *Brucella* spp. are fastidious bacteria, and their intracellular niche requires specific metabolic adaptation to nutrient deficiency. Hence, classical brucellae grow slowly and exhibit limited metabolic activity. In the last decade, novel *Brucella* strains have been identified which can be differentiated from the classical species by atypical phenotypic features, e.g., LPS variants [12,13], motility [14], higher metabolic activity [15] or modified metabolism [16,17], and fast growth [18,19]. Using common microbiological methods, these characteristics have often led to misidentification as *Ochrobactrum anthropi* or *Ochrobactrum intermedium* [5,20,21,22,23]. Most species of the former genus *Ochrobactrum* are environmental bacteria, which may cause opportunistic infections in humans [24]. The novel *Brucella* spp. that have been described so far are *Brucella microti* (prevalent in common voles, red foxes, and wild boars) [15,22,25], *Brucella inopinata* (in humans) [21,26], *Brucella vulpis* (in red foxes) [16], and *Brucella papionis* (in baboons) [17].

*B. microti* was the first atypical species which was isolated from soil samples [27] and revealed similarities to phytopathogens such as *Agrobacterium* spp. and plant symbionts such as *Rhizobium* spp., all belonging to the class of Alphaproteobacteria [19,28]. Environmental persistence of the atypical *Brucella* species and the increasing variety of reservoir hosts may facilitate their epizootic spread. Rodents, omnivores, and soil, for instance, may constitute an enzootic transmission cycle supporting long-term survival of *B. microti* in the natural environment [19,22,27]. This open lifestyle is in contrast to the intracellular evolution of classical *Brucella* species, which is limited to only a few specific hosts. Since the genome sequences of *B. microti* CCM 4915 and *B. suis* 1330 were found to be 99.84% identical in aligned regions [29], some of their phenotypic differences may result from species-specific gene expression and functionality [19,30].

*B. inopinata* (BO1) [21,26] and a similar strain (BO2) [31] were primarily identified as the cause of opportunistic infections in humans, presenting as a wound abscess of a breast implant and chronic destructive pneumonia, respectively. The primary animal or environmental reservoir of these two strains is still unknown. However, strains isolated from wild native rodent species in North Queensland, Australia, were assumed to be new lineages of *B. inopinata* [31]. *B. inopinata* is a fast growing and metabolically very active species comparable to *B. microti* [19,21]. *B. vulpis* is characterized by a limited metabolic activity comparable to that of classical species, especially with respect to the utilization of sugars, but which also shows atypical traits such as negative nitrate reductase and oxidase reactions [32]. In phylogenetic analyses, *B. vulpis* formed a novel, but rather distant, clade of the hitherto called classical species [10].

In recent years, more and more atypical *Brucella* spp. have emerged from cold-blooded animals. *B. inopinata*- and *B. microti*-like strains were found in amphibians, primarily in African bullfrogs [14,33], and later on in various other frog species all over the world [5,34,35,36,37,38] as well as toads [23,39,40,41], reptiles such as the panther chameleon (*Furcifer pardalis*) [42], and fish, such as the bluespotted ribbontail ray (*Taeniura lymma*) [43]. A first human case caused by an amphibian-type *Brucella* spp. has been recently reported in France [44]. Since some of the novel and atypical *Brucella* spp. exhibit either a modified O-polysaccharide moiety, which is the immunodominant subunit of the smooth lipopolysaccharide (LPS), or a rough LPS, serological diagnosis of these infections may be compromised [13]. Hence, the role of the novel *Brucella* spp. as a zoonotic pathogen cannot yet be assessed due to a lack of adequate diagnostic tools both for animals and humans.

While genome similarities among classical and atypical species are still high (ANI > 95%) [23,32], the genomes of the latter are more heterogenous (Figure 1) and also include genetic information found in environmental bacteria [10]. This phenomenon might be explained by genome size reduction in the more specialized classical brucellae when compared to former *Ochrobactrum* spp. and/or by horizontal gene transfer between the novel species and other soil-associated bacteria.

Wildlife and the natural environment constitute the reservoir of the new and atypical *Brucella* spp. [45], which makes surveillance and control of these potentially epizootic or zoonotic pathogens a challenge. The genetically divergent species are considered as ancient ancestors of the classical human pathogenic *Brucella* spp., but their relevance for animal and public health is unknown. In our review, we give an overview of the current knowledge on the new and atypical *Brucella* species with special focus on their phylogenetic placement in the genus, certain metabolic features, acid stress resistance, and their behaviour in well-established infection models. Finally, we make a suggestion for the terminology that should be consistently applied to describe the novel *Brucella* species.

## 2. Genomics and Phylogeny

### 2.1. Classification Criteria for Brucella spp.

This section focuses on phylogeny and genomics of the heterogeneous group of novel, frequently called atypical *Brucella* spp. that encompass species and isolates with diverging phenotypes and/or genotypes in comparison to the hitherto classical species. For a comprehensive overview on how molecular approaches have helped to shape the taxonomy of the genus *Brucella* with specific emphasis on the phylogeny of classical *Brucella*, readers are referred to other sources [10,28,46]. Some of the novel strains are more genetically diverse (*B. inopinata*, isolates from amphibians and Australian rodents) in comparison to the core *Brucella* species, and thus form clades that diverged early relative to the core clade (Figure 1). Others have a high degree of genetic homology (*B. microti*, *B. papionis*) but differ in biotyping when compared to the hitherto classical *Brucella* species [10]. The latter are therefore part of the core clade but show diverging phenotypes with respect to metabolic activity (see also Section 3. Metabolism), growth rate, motility, phage lysis, dye sensitivity, and/or serum agglutination. In addition, the host range of the novel brucellae reaches beyond mammalian hosts, including vertebrates like amphibians, reptiles, and fish, but they can also be found in the environment [27,35,43].

It should be noted that a large-scale reevaluation of genomic data has recently led to taxonomic reclassification of the genus *Ochrobactrum*. These former *Ochrobactrum* spp. now build a heterogeneous basal clade within the genus *Brucella*, distant from the novel and core *Brucella* species (Figure 1) [4]. Hence, generalized statements on *Brucella*, for instance that IS*711* is a genus-specific marker (see below), do not apply to these former *Ochrobactrum* spp., including their most prominent—at least in the *Brucella* research field—representatives, *O. intermedium* and *O. anthropi*. For these species, the former nomenclature has been used throughout this review for ease of comprehension.

Before merging *Brucella* and *Ochrobactrum* into a single genus, attribution to the genus *Brucella* was readily achieved by polymerase chain reactions (PCRs) targeting the repetitive insertion sequence IS*711* (also known as IS*6501*) [47,48]. Frequency and location of IS*711* yield mostly unique patterns contributing to the discrimination of *Brucella* on an inter-species level, thus called IS*711* fingerprinting [49,50]. Several multiplex PCRs (e.g., AMOS—*B. abortus*, *B. melitensis*, *B. ovis*, *B. suis*—PCR, and the Bruce-ladder PCR) have been established to discriminate a set of known core *Brucella* species, and have been adapted to new needs in the developing *Brucella* field (see below) [51,52].

DNA–DNA-hybridization (DDH) used to be the gold standard in prokaryotic species delineation, but this technique, with its proposed 70% DNA-relatedness threshold [53], posed an issue for the highly homologous *Brucella* species [54]. Determination of the ANI with species boundaries of 95–96% ANI (a modern DDH alternative) of gene sequences, core, or whole genomes (cg or wg) can, similarly to DDH, indicate whether an isolate fits within the genus (ANI of >99% for classical *Brucella*) [55,56]. Thus, this in silico approach provides valuable information on genomic homology.

Sequencing of phylogenetic markers helps to discriminate novel species and strains. Conserved genes such as those encoding 16S rRNA and *recA* are almost identical in all classical brucellae but contain nucleotide polymorphisms in novel species [23]. Genotyping approaches using whole genome sequences or specifically amplified loci following molecular typing methods such as multilocus sequence analysis (MLSA), multiple-locus variable-number tandem repeat (VNTR) analysis (MLVA), or single-nucleotide polymorphism (SNP) analysis have been extremely useful to determine the genetic diversity among *Brucella* spp.

### 2.2. Novel Brucella spp. as Part of the Core Clade

**Figure 2:**
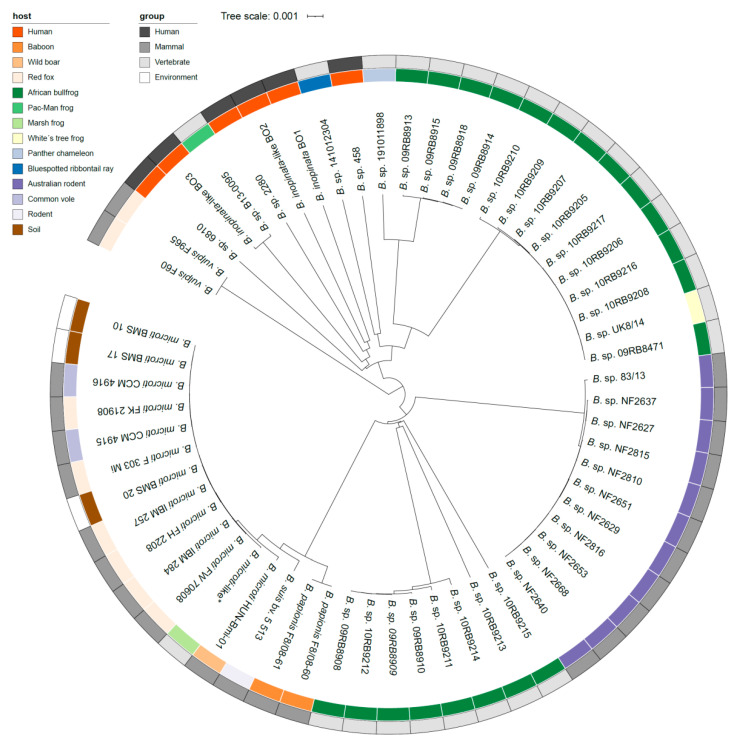
Circular tree illustrating the phylogenetic relationship of novel *Brucella* isolates. The phylogenetic tree was visualized with iTOL (v6) and is based on pairwise analysis of average nucleotide identities (fastANI v1.32) [5] and hierarchical clustering (UPGMA method). Publicly available genome sequences retrieved from the NCBI database (see Data Availability Statement) were complemented with own unpublished genome sequences. Colored rings indicate the host/environment where the strain was originally isolated from. From inside to outside: ‘host’ and ‘group’, presenting the exact host/environment and a rough grouping, respectively. The group ‘Vertebrate’ excludes mammals, and the group ‘Mammal’ excludes humans. *: *B. microti*-like strain 17-2122-4144.

Isolates from the common vole (*Microtus arvalis*) were affiliated to the genus *Brucella* based on phylogenetic markers—16S rRNA and *recA* gene sequences and IS*711*—while these strains displayed diverging phenotypes in comparison to the fastidious and highly homologous classical *Brucella* spp. [15,20]. These unique isolates, CCM 4915—now the type strain—and CCM 4916, were thus considered representatives of a new species, *B. microti* [15,20]. IS*711* fingerprinting revealed a unique pattern, not found in other *Brucella*. Additionally, a novel 12-kb genomic island, a bacteriophage-related insertion absent in all *Brucella* species described so far, was identified [27,29]. The outer membrane protein locus *omp2* of *B. microti* (CCM 4195) is most similar to that of *B. suis* bv. 5 (strain 513), with one and two nucleotide differences in *omp2a* and *omp2b*, respectively [19]. Interestingly, *B. suis* bv. 5 strain 513, a wild rodent isolate, groups apart from the other *B. suis* biovars (bv. 1–4) and shows high homology to *B. microti* isolates that were all collected in Central Europe [28,57]. Furthermore, *B. suis* bv. 5 strain 513 forms a sister clade to the latter species (Figure 1 and Figure 2). MLSA and MLVA positioned the *B. microti* cluster apart from the other core *Brucella*. Since their initial description, *B. microti* strains have been isolated from soil [19,27], red foxes (*Vulpes vulpes*) [19,25], and wild boar (*Sus scrofa*) [22], and *B. microti*-like strains have been isolated from domestic marsh frogs (*Pelophylax ridibundus*) (Figure 2) [5,45]. The Bruce-ladder multiplex PCR, which identified these strains as *B. suis* in its original design [15], was adapted through making use of the unique 12 kb genomic island described before. This adaptation resulted in an additional 510 bp fragment [58], allowing identification of all isolated *B. microti* and *B. microti*-like strains to date [5,19,22,25,27]. Despite their various origins, all *B. microti* isolates, except for strain HUN-Bmi-01 from wild boar and strain 17-2122-4144 from a marsh frog, possess identical 16S rRNA, *recA*, and *omp2* gene sequences, as well as identical MLSA profiles at 21 loci (MLSA-21) [19]. Based on genome sequence analysis, strain HUN-Bmi-01 contains 30 SNPs in orthologous loci when compared to the reference strain CCM 4195 [22]. In contrast to the majority of amphibian *Brucella* isolates (see below), the frog strain 17-2122-4144 is taxonomically grouped close to *B. microti* and more distant from the *B. inopinata* clade (Figure 2), which is why it is designated as *Brucella microti*-like [5]. This affiliation is based on several molecular approaches, including analysis of the *recA* and 16S rRNA gene sequences, as well as wgSNPs.

The species *B. papionis* is constituted of two strains (F8/08-60 and F8/08-61) isolated from baboons (*Papio* sp.) [17]. The strains have identical 16S rRNA gene sequences with two nucleotide mutations when compared to the classical *Brucella* species. In addition, these isolates share an identical high-copy-number IS*711* profile with several baboon isolate-specific IS*711* copies [17]. Phylogenetically, *B. papionis* groups close to *B. ceti*, *B. pinnipedialis*, and *B. ovis*, and apart from *B. melitensis* and *B. abortus* (Figure 1). Furthermore, the *omp2* gene loci of *B. papionis* and *B. ceti* consist of two almost identical *omp2b* gene copies [17]. According to WGS data, *B. papionis* shares a common ancestor with *B. ovis* [17].

### 2.3. Novel Brucella spp. as Ancient Ancestors

Two isolates from red foxes (*Vulpes vulpes*) containing *bcsp31* and IS*711* gene sequences were considered a novel species, later named *B. vulpis* [16,32]. The respective strains—F60 and F965—share an average ANI of >97% with the *Brucella*-type species (*B. melitensis* 16M) [32]. Phylogenetic marker genes encoding 16S rRNA and *recA* differ in three and ten nucleotides, respectively, when compared to the *Brucella* consensus sequence [16]. Furthermore, *B. vulpis* has a unique high-copy IS*711* fingerprint and its *omp2* locus contains two *omp2a* gene copies [32]. Interestingly, the genome of *B. vulpis* contains sequences that are not present in classical *Brucella* and might have been acquired by horizontal gene transfer in a yet unidentified soil reservoir. These sequences include ABC transporters and genes with metabolic function known from soil-associated bacteria, for instance from the former *Ochrobactrum* and *Rhizobium* [32]. *B. vulpis* is distinct from the core clade and is closely associated to the heterogeneous species and strains that are introduced next (Figure 1 and Figure 2).

The first strain isolated from a brucellosis patient that displayed genetic divergence when compared to the core *Brucella*, and thus forms a distinct but closely related lineage, is *B. inopinata* BO1 [21,26]. The *B. inopinata* strains BO1 and BO2, another human isolate that clusters apart from the core clade [31], share a unique IS*711* multi-copy fingerprint discriminating them from other *Brucella* spp. Both strains contain unique mutations in comparison to the *Brucella* 16S rRNA consensus sequence and mutations in the *recA* gene sequence that do not impact protein structure or function [31]. MLSA-9 supports the positioning of BO1 and BO2 relative to the core *Brucella* [26,31]. Furthermore, MLVA-15 and *recA* gene analysis identified unique VNTRs and nucleotide substitutions, respectively, separating BO1and BO2 from the core clade and from one another [31]. The *omp2* gene loci of strain BO1 and BO2 are alike and cluster with the atypical *B. suis* strain 83-210 from an Australian rodent [31,59]. Primary animal or environmental reservoirs of *B. inopinata* strains have not yet been verified. Phylogenetically, the *B. inopinata* strains BO1 and BO2 form a diverse clade containing several Australian rodent strains isolated decades ago and dozens of new amphibian strains, as well as a reptile and a fish strain isolated in recent years. This grouping is supported by SNP analysis [33,35,36,43]. The clade is basal to the core *Brucella* and distantly related to the former *Ochrobactrum* clade (Figure 1).

The seven Australian rodent strains—originally misidentified as *B. suis* bv. 3—contain the same *B. inopinata*-specific nucleotide substitutions in the 16S rRNA gene sequence [60,61]. Nevertheless, these Australian rodent isolates display large diversity determined by MLVA analysis, which identified unique types for each isolate [61]. Remarkably, another Australian rodent isolate (83-13) also showed a high degree of genetic similarity to the *B. inopinata* strains (Figure 2) [61].

*Brucella* spp. have been isolated from several different amphibian hosts. The African bullfrog (*Pyxicephalus edulis*) strains, for instance, comprise two groups based on 16S rRNA sequences. The sequence of the one group is identical to the *B. inopinata* consensus sequence, whereas the sequence of the other group contains a 44-nucleotide insertion of *Ochrobactrum* origin [14]. Furthermore, they have unique MLSA types and are split in several lineages (Figure 2). The strain B13-0095 from a Pac-Man frog (*Ceratophrys ornata*) shares 100% 16S rRNA sequence identity with *B. inopinata* strains [36]. Furthermore, two genomic regions that contain metabolic features originating from soil bacteria—conserved in former *O. anthropi*—are present in strain B13-0095 and are absent in core *Brucella* [36]. One gene cluster enables ectoine utilization (see Section 3) by strain B13-0095. The other region, organized similarly to a plasmid, is also present in *B. inopinata* BO1 and strain BO2, and enables uptake and utilization of rhamnose [36] (see also Section 3), which is a product of pectin polysaccharide degradation by saprophytic bacteria [62].

In a recent case report, a patient frequently and directly exposed to exotic animals, including amphibians and rodents, was diagnosed with brucellosis [44]. *Brucella* was successfully isolated from cervical lymph nodes. The strain was named *B. inopinata*-like 3 (BO3), since it clusters together with the distinct clade of novel *Brucella* consisting of *B. inopinata* strains BO1 and BO2 as well as frog isolates; however, BO3 was most similar to the Pac-Man frog strain B13-0095 (Figure 2) [44]. Furthermore, from our NCBI database query, we retrieved three sequences of recent clinical isolates—*B*. sp. 2280 (BioSample: SAMN12091575), *B*. sp. 6810 (BioSample: SAMN15962648), and *B*. sp. 458 (BioSample: SAMN18395631)—that all cluster close to or within the *B. inopinata* clade that also contains some of the African bullfrog isolates (Figure 2). These clinical isolates originated from human lymph nodes, blood, and lung tissue, and were associated with brucellosis and granuloma formation, respectively.

In conclusion, the majority of novel *Brucella* that have been isolated over the past 15 years comprise genetically heterogeneous strains that form the relatively diverse non-core clade, basal to the genetically highly homogeneous core clade including only two novel species.

## 3. Metabolism

### 3.1. General Physiological Characteristics of Brucella spp.

**Figure 3:**
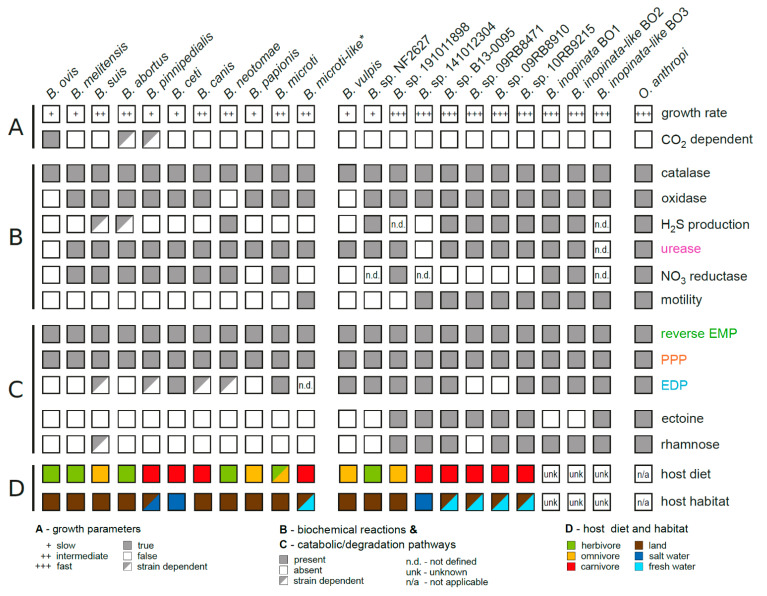
Characteristics of classical and novel *Brucella* strains and species. Shown are the phenotypic properties of *Brucella* species and strains, including former *O. anthropi* (**A**,**B**), the presence or absence of metabolic pathways in *Brucella* (**C**), and the typical habitats and diets of *Brucella* host organisms (**D**). *: *B. microti*-like strain 17-2122-4144.

Novel, as well as classical, *Brucella* isolates are chemoorganotrophic, aerobic bacteria harboring catalase and cytochrome c oxidase activities, the latter being absent in *B. ovis* and *B. neotomae* (Figure 3). The catalase of *Brucella* sp. participates together with the peroxiredoxin AhpC in detoxifying endogenous hydrogen peroxide (H_2_O_2_) generated during aerobic respiration [63,64], and also provides protection against the oxidative defense mechanisms of the host immune system, especially during entry of *Brucella* into macrophages.

Likewise, H_2_S, which is produced by certain classical *Brucella* and most novel *Brucella* isolates (Figure 3), may support an important self-protective mechanism through scavenging harmful oxidants [65,66], in particular the reactive oxygen species (ROS) superoxide anion (O_2_^−^) and H_2_O_2_, generated by phagocytic immune cells to combat *Brucella*. H_2_S generation is facilitated by the uptake of sulfate, its assimilatory reduction to sulfite, and subsequently to H_2_S, which can be released or alternatively used by *Brucella* for the conversion of serine into cysteine, the latter contributing to the peroxide stress defense and survival inside macrophages [67]. Furthermore, H_2_S might be released through a 3-mercaptopyruvate sulfurtransferase SseA during cysteine degradation [68], when *Brucella* persists in an environment with sufficient proteinaceous, cysteine-containing substrates. Future studies have to elucidate whether H_2_S produced by *Brucella* might also interfere as a “small molecule signaling agent” with the host physiology and immune response during infection [69,70].

While establishing a replicative niche within its hosts, *Brucella* is not only exposed to ROS but also to the damaging effects of reactive nitrogen species (RNS), especially the nitric oxide (NO) that is produced by the deamination of arginine through the inducible nitric oxide synthase (iNOS) in macrophages [71]. Arginine utilization is an intra- and interspecies variable trait in *Brucella* [72,73], whereby arginine-catabolizing strains might reduce the host nitric oxide production by competing for arginine with NO-producing cells [74]. In addition, *Brucella* encodes protective enzymes against RNS stress, such as NorB and NorD [75,76], catalyzing the reduction of NO to gaseous nitrous oxide. NO can also react with ROS to nitrate (NO_3_^−^) [77], which serves as an alternative electron acceptor in oxygen-independent respiration [78]. The ability to reduce nitrate is widespread in bacteria as well as in various *Brucella* species (Figure 3) and promotes the growth and persistence of *Brucella* in the oxygen-limited environment of host tissues [75,78].

With the exception of *B. ovis*, classical and novel *Brucella* isolates, as well as former *O. anthropi* and *O. intermedium*, exhibit urease activity. Urease converts urea to CO_2_ and ammonia, thereby contributing to the acid resistance of *Brucella* and promoting host infection by enhancing the viability of the bacteria during their exposure to gastric acid. This is stressed by the observation that closely related *Bartonella* with its blood-borne transmission lost the urease function [79]. However, host-independent, environmental bacteria also harbor urease genes, reflecting the importance of urease for the nitrogen balance of bacteria through the interconnection with an intact urea cycle, found in *Brucella* as well [80]. Especially, when *Brucella* spp. are grown on glutamate or asparagine, a significant amount of arginine is produced and subsequently hydrolyzed by the conserved arginase into urea and ornithine [81].

### 3.2. Growth Properties of Novel versus Classical Brucella

*Brucella* spp. have initially been described as fastidious organisms that require demanding cultivation conditions in vitro with nutritionally complex media based on peptones and preferentially supplemented with blood or serum [82]. However, early studies examining the growth of classical *Brucella* species in defined media revealed that most strains are able to proliferate in less complex media [83], only consisting of salt solutions, ammonia as a nitrogen source, single carbon sources and a restricted number of vitamins, especially biotin [84]. *B. microti*, the first reported atypical *Brucella* species, shows accelerated growth kinetics when compared to classical *Brucella* species (Figure 3). This phenomenon was also described for *B. inopinata*(-like) strains from humans and a variety of *Brucella* strains isolated from frogs (Figure 3), and correlates with extended nutrient substrate utilization patterns as well as enzyme activities [72], resembling the growth characteristics of the ubiquitous former *O. intermedium* and *O. anthropi*. However, fast growth is not an adequate trait to discriminate between classical and novel *Brucella* species, since *B. vulpis* and *Brucella* strains isolated from Australian rodents show growth dynamics similar to those of *B. melitensis* or *B. abortus* (Figure 3), whereas the proliferation kinetics of the *B. suis* bv. 5 strain 513 resembles that of the fast-growing atypical *Brucella* and former *Ochrobactrum* species [85].

In general, the widely used commercial systems for the biochemical testing of bacteria, namely API 20NE, RapID NF Plus, and VITEK 2, have revealed pitfalls in discriminating classical and in particular atypical *Brucella* isolates from primarily environmental (former) *Ochrobactrum* spp. [23,86]. However, comprehensive phenotypic characterization shows striking intra- and inter-species variability in the substrate utilization patterns of *Brucella* [19,72,87]. Since novel *Brucella* isolates are also facultative intracellular pathogens that survive nutritional restrictions within host macrophages [14,18,36,88], such physiological differences within the genus may mirror the flexible adaptability of *Brucella* species to their different hosts through genetic micro- and macroevolution [89]. Animals, with their distinct gastrointestinal physiology and diets (herbivores, carnivores, omnivores) (Figure 3), harbor different microbiota that may interact with *Brucella* and compete for nutrients following oral infection. Moreover, the host animals of *Brucella* have different thermophysiologies and belong to the group of homeotherms (mammals) or poikilotherms (rays, amphibians, reptiles), which may result in additional adaptation mechanisms of the different *Brucella* species. The extended substrate utilization patterns of certain novel *Brucella*, similar to that of the ubiquitous environmental *Ochrobactrum* species, further suggests an improved adaptation of these bacteria to the persistence and growth outside their hosts in nutritionally diverse soil or aquatic surroundings that represent the habitats of rodents, reptiles, and amphibians. Although growth outside hosts has not been proven yet, this hypothesis is further supported by the fact that *Brucella* strains isolated from frogs and former *Ochrobactrum* are flagellated and motile (Figure 3) [14,36], which promotes enhanced chemotaxis towards nutrients in the environment [90].

### 3.3. Atypical Brucella spp. Are Characterized by a Diversified Metabolism and Expanded Nutrient Utilization When Compared to Classical Species

Based on the very high DNA homology among its species, it has been suggested for some time to consider *Brucella* as a monospecific genus [54]. Comparative genomics analyses between species has revealed a high level of preservation in gene content and synteny [91]. Yet, genetic micro- and macrodiversity shaped the evolution of this genus into different species [28], which are adapted to colonize different hosts by the acquisition and modification of distinct metabolic and virulence properties [89,92]. In particular, the loss of gene functions through pseudogenization by SNP and insertions of transposons, as well as the acquisition or loss of genomic islands and plasticity regions drive the metabolic diversification of *Brucella* [89,93,94,95,96,97,98]. Further comparative genomics analyses will clarify as to whether distinct distribution patterns of metabolic genes in certain *Brucella* species reflect a clear host adaptation, as seen for *Campylobacter jejuni* lineages colonizing cattle [99].

#### 3.3.1. Intra- and Interspecies Variation in the Central Carbon Catabolism of Brucella

**Figure 4:**
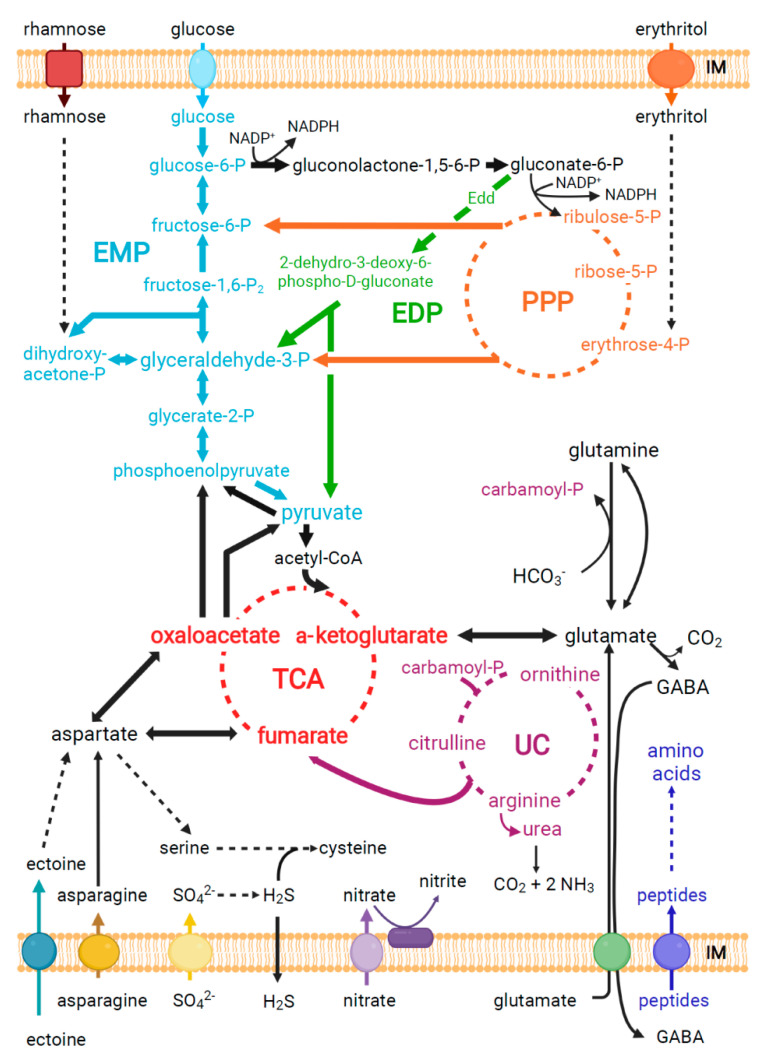
Metabolic pathways of *Brucella*. Major metabolic pathways of *Brucella* and examples of substrates fueling this metabolic network, as described in Section 3. The pentose phosphate pathway (PPP), the Embden-Meyer-Parnas (EMP) pathway, the tricarboxylic acid (TCA) cycle, and the urea cycle (UC) are conserved in *Brucella*. Only a subset of *Brucella* species harbor an active Entner-Doudoroff pathway (EDP) that correlates with an active phosphogluconate dehydratase (Edd) enzyme converting gluconate-6-phosphate into 2-dehydro-3-deoxy-6-phospho-D-gluconate. Solid lines represent direct substrate conversion by a single enzymatic reaction, whereas dashed lines illustrate multistep conversions of substrates with the involvement of several, subsequent enzymatic reactions. Transporters involved in translocation of indicated substrates from the periplasm across the inner membrane (IM) into the cytoplasm of *Brucella* are displayed schematically. Acetyl-CoA—acetyl coenzyme A; GABA—gamma-aminobutyric acid; CO_2_—carbon dioxide; HCO_3_^−^—bicarbonate; H_2_S—hydrogen sulfide; NADP—Nicotinamide adenine dinucleotide phosphate; NH_3_—ammonia; SO_4_^2−^—sulfate. Created with BioRender.com.

Besides all genetic and phenotypic variability, the genetic makeup of the central metabolic framework that fuels the growth of classical and novel *Brucella* has been suggested to be conserved in all species [100]. A complete pentose phosphate pathway (PPP) facilitates the catabolic and anabolic utilization of hexoses, pentoses, and the sugar alcohol erythritol as energy sources and precursors for the biosynthesis of nucleotides and amino acids (Figure 4) [101,102]. Importantly, the oxidative branch of the PPP provides, in contrast to the Embden-Meyerhof-Parnas (EMP) pathway, nicotinamide adenine dinucleotide phosphate (NADPH), which can be used as reducing equivalents [103]. This electron donor is necessary for the de novo synthesis of biomolecules, especially during persistence in nutrient-poor niches such as the *Brucella*-containing vacuole, and for the protection against oxidative stress as described for *Francisella* [104]. The reductive branch of the PPP feeds trioses into the lower, well-conserved part of the EMP pathway, and subsequently fuels the active and complete tricarboxylic acid (TCA) cycle of *Brucella*, generating NADH (Figure 4) [100]. Due to a missing 6-phosphofructokinase (PFK) gene, the upper ATP-consuming part of the glycolytic EMP pathway is non-functional in classical and novel *Brucella* species, including the former *O. anthropi* and *O. intermedium*. Such an interrupted upper EMP pathway resulting from a lack of the *pfk* gene can also be found in environmental, non-pathogenic, and other pathogenic bacteria such as *Helicobacter pylori* [105], *C. jejuni* [106], *Neisseria meningitidis* [107], as well as other Alphaproteobacteria, e.g., *Bartonella* *henselae* [108] or *Zymomonas mobilis* [109]. However, all these pathogens and the *Brucella* species encode the enzyme fructose 1,6-bisphosphatase that catalyzes the conversion of fructose 1,6-bisphosphate to fructose 6-bisphosphate, and therefore make use of a complete reverse EMP pathway for gluconeogenesis. This indicates the importance of the gluconeogenic nature of the EMP pathway for *Brucella*, especially during host infection [110], as seen for other pathogenic bacteria and protozoa such as *Francisella* or *Toxoplasma gondii* [111,112]. Interestingly, the slow-growing and less virulent *B. ovis* harbors a frameshift mutation in the phosphoenolpyruvate carboxykinase *pckA* gene, negatively affecting gluconeogenesis, since oxaloacetate cannot be converted into phosphoenolpyruvate [95].

The best-characterized *Brucella* strains belong to the classical species and catabolize carbohydrates exclusively through the PPP [100]. However, a recent study showed that some *Brucella* isolates harbor, in addition to the PPP, an active Entner-Doudoroff pathway (EDP) (Figure 3 and Figure 4), the latter being mainly responsible for the glucose catabolism in the environmental Alphaproteobacteria such as *Agrobacterium*, *Caulobacter*, and *Rhizobium* [113]. The inactive EDP in the classical strains *B. melitensis* bv. 1 strain 16M, *B*. *abortus* bv. 1 strain 2308, and *B. suis* bv. 1 strain 1330 is caused by a nonsynonymous point mutation in the 6-phosphogluconate dehydratase gene *edd*, leading to an A178P amino acid exchange in Edd, or by a frameshift mutation leading to a truncated gene [113]. In contrast, isotopologue profiling and genetic experiments revealed an active Edd in novel *B. microti* and *B. inopinata* BO1, but also in *B. neotomae* and *B. suis* bv. 5 strain 513 [113,114]. The redundancy in substrate utilization by the PPP and EDP in *B. microti* and *B. suis* bv. 5 strain 513 is illustrated by infection experiments with mice, since mutants in one of these pathways did not show colonization defects, in contrast to infection experiments with a PPP-mutant of *B. suis* bv. 1 strain 1330 [113,114]. Based on these findings, the genus *Brucella* can be divided into two classes with respect to carbohydrate catabolism: the first class relies on PPP only for glucose catabolism, whereas the second class catabolizes glucose via PPP and EDP, representing the transition from the environmental Alphaproteobacteria, relying solely on EDP, to the highly host-adapted *Brucella* species using only PPP for glucose metabolism [113]. Interestingly, protein sequence comparisons of *Brucella* Edd enzymes reveal inter- but also intra-species variation (unpublished data): While the nonsynonymous mutation A178P is commonly found in Edd of *B. melitensis* and *B. abortus*, active as well as inactive forms of Edd are found in *B. suis*, *B. canis*, and *B. pinnipedialis*, whereas *B. ceti*, *B. vulpis*, *B. inopinata* BO2, *Brucella* sp. 191011898, and most *Brucella* isolates from frogs produce an active Edd. The slow-growing *B. ovis* isolates harbor truncated Edd proteins.

#### 3.3.2. Macro- and Microdiversity Shape the Nutrient Utilization of Brucella

Traditional biochemical characterization and growth experiments revealed striking inter- and intraspecies diversity of substrate utilization by *Brucella*, often preventing their unambiguous classification into species or even biovars [115]. Various studies demonstrated that the higher growth rates of atypical *Brucella* correlate with an enhanced metabolic activity and substrate utilization [72]. A comprehensive, semi-automated phenotypic profiling for peptidase-, glycosidase, phosphatase, and esterase activities showed that most of the classical and novel *Brucella* isolates, as well as the former *O. anthropi* and *O. intermedium*, have similar peptidase activities, whereas more pronounced glycosidase activities could be observed in the atypical *B. microti*, *B. inopinata*, as well as in former *O. anthropi* and *O. intermedium*, than in classical *Brucella* species [72]. In general, amino acid and carbohydrate utilization is more prominent in atypical *Brucella* species than in classical species [72], whereby minor variations occur in the catabolism of main carbohydrates such as glucose, arabinose, galactose, xylose, or erythritol.

##### Microdiversity

The differences in peptide, amino acid and carbohydrate metabolism can be mainly traced back to different sets of active substrate transporters in *Brucella* species, but also to point mutations in genes of catabolic pathways. In *B. ovis*, *ery* genes required for the uptake and catabolism of erythritol, the glucose/galactose transporter gene *gluP*, genes of a predicted ribose ABC transporter, and further genes of carbohydrate ABC transporters with unknown substrate specificity are pseudogenes [95]. In addition, there are intra- and interspecies variations in substrate-binding proteins, such as the periplasmic peptide binding protein of *B. melitensis* (BMEI0433) which is also conserved in *B. microti* (BMI_I1599) but truncated in most *B. abortus* isolates [80], in various *B. suis* isolates, and in *B. vulpis*. SNPs are responsible for growth alterations such as the CO_2_-dependent growth of *B. ovis*, certain *B. abortus* biovars and *B. pinnipedialis* strains, caused by a defective carbonic anhydrase [116]. Furthermore, the β-ketoadipate pathway involved in the degradation of the lignin-derived β-ketoadipate to succinyl- and acetyl-CoA is not active in all *Brucella* species. While *O. anthropi* harbors a 12-genes operon comprising an active β-ketoadipate pathway, which is also active in *B. microti*, the β-ketoadipate pathway in the classical species *B. melitensis*, *B. abortus*, and *B. suis* has been inactivated by mutations in individual pathway genes or through the deletion of the complete operon, as in *B. suis* strain 23445 [29,97]. Some environmental Alphaproteobacteria, such as *Sinorhizobium meliloti*, benefit from this pathway once a variety of fungi and soil bacteria have started to degrade the aromatic polymer lignin, which is a substantial component of plant cell walls [117].

##### Macrodiversity

A striking difference between classical *Brucella* species and the *Brucella* sp. B13-0095 isolate from a Pac-Man frog is the ability of the latter to use ectoine as growth substrate [36]. This cyclic amino acid functions in ectoine-producing bacteria as solute to cope with osmotic stress and under extreme temperatures [118]. Strain B13-0095 and other atypical *Brucella* species may have lost the genes required to synthesize ectoine, whereas various Alphaproteobacteria in aquatic habitats harbor anabolic and catabolic ectoine genes [119]. Ectoine-utilizing *Brucella* isolates (Figure 3) contain a 12.5 kb plasticity region that encodes an ectoine uptake transporter and enzymes transforming this amino acid to aspartate, which fuels the TCA cycle and is a precursor for the biosynthesis of isoleucine, lysine, methionine, and threonine [36]. Since certain bacteria release ectoine under changing environmental conditions, atypical *Brucella* might benefit from such an ectoine cross-feeding [120].

A plasticity region coding for genes of a rhamnose catabolism pathway has also been first described for the *Brucella* strain B13-0095 isolated from a Pac-Man frog, as well as for *B. inopinata* strains BO1 and BO2, with best homologies to orthologous genes in former *O. anthropi* [36]. Growth experiments suggested that the utilization of rhamnose as a sole carbon source is restricted to these new and atypical *Brucella* strains. Genes involved in rhamnose catabolism can also be found in *Brucella* isolates from a panther chameleon and a bluespotted ribbontail ray, and more surprisingly in *B. suis* bv. 4 strain 40 (unpublished data) (Figure 3). Hence, distinct metabolic traits are mosaically dispersed throughout the genus *Brucella* by horizontal gene transfer and successively fine-tune the metabolic adaptation of these bacteria to specific niches in different hosts, independently of the phylogenetic position of strains and species. Deoxy-hexose rhamnose is commonly found in the structural polymers of plant cell walls or in the pectin of fruits. Hence, former *O. anthropi, B*. *microti*, and other atypical *Brucella* species able to persist in the environment might encounter rhamnose through the degradation of plant material. Moreover, rhamnose is also produced as major carbohydrate in fresh and sea water microalgae together with mannose, xylose, glucose, and fucose [121], and the decay of these microalgae might provide rhamnose and other carbon sources for *Brucella* associated with hosts that inhabit aquatic environments, such as amphibians or fish.

In summary, most classical and atypical *Brucella* share the metabolic traits necessary to overcome the oxidative and pH stresses that they encounter during infection of their primary host organisms. Hence, the novel *Brucella* species are also able to counteract these initial host defense mechanisms. However, phenotypic characterization has revealed a surprising metabolic diversity among classical and atypical *Brucella*, despite the high genetic homogeneity within the genus. Most strikingly, the additional EDP activity in certain *Brucella* isolates illustrates the variance in the central carbon metabolism of this genus and might reflect the specific adaptation to different ecological niches and environmental conditions.

Although the EDP provides less ATP than the EMP, it might be especially beneficial for *Brucella* that better persist in the environment or have a less extensively host-adapted lifestyle than e.g., *B. abortus* or *B. melitensis*. The EDP is thermodynamically more favorable and requires fewer resources for the synthesis of enzymatic proteins when compared to the EMP [122]. Since all classical and atypical *Brucella* have an inactive EMP, the EDP complements the cyclic PPP, which is active in all *Brucella* as the main glucose utilization pathway and provides energy, NADPH, and precursors for biosynthetic reactions. The *Brucella* strains that are EDP-positive belong to the growing number of mainly environmental bacteria that catabolize carbon sources via this pathway, which is used solely or in combination with the PPP, especially in aerobic or in facultative anaerobic bacteria [122].

## 4. Adaptation to Acid Stress

Due to its lifestyle as a foodborne, usually orally transmitted, and facultative intracellular bacterium, *Brucella* may encounter acid stress in various environments, including soil, fermented food, the gastro-intestinal tract, and, last but not least, the intracellular compartments of animal reservoir hosts and humans. The major possible deleterious effects of increased proton concentrations for the bacterium are reduced enzymatic activity, protein unfolding, and damage of cell membranes and DNA [123].

### 4.1. Responses to Extreme Acid Stress

**Figure 5:**
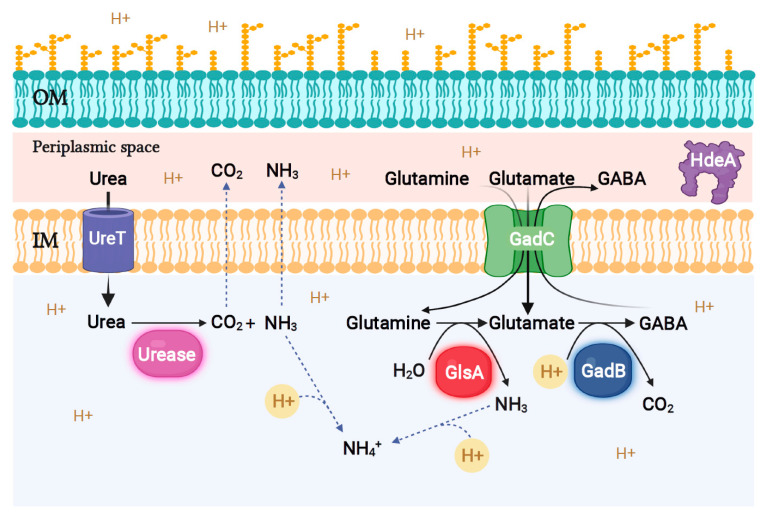
Schematic representation of extreme acid resistance mechanisms described in *Brucella*. The urease system, made up of urease and urea transporter UreT, imports and hydrolyzes urea, leading to the production of ammonia (NH_3_) which, by capturing a proton (H^+^), is converted into ammonium (NH_4_^+^). The glutamate-dependent acid resistance (GDAR) system consists of two proteins: the cytoplasmic glutamate-decarboxylase GadB, and the L-glutamate (Glu)/gamma-aminobutyrate (GABA) membrane antiporter GadC. H^+^ entry reduces intracellular pH and activates GadB that consumes H^+^ by converting glutamate into GABA. The GadC antiporter feeds the GDAR system by exchanging charged GABA with extracellular Glu. The glutamine-dependent system consists of the L-glutamine (Gln)/Glu antiporter GadC and the glutaminase GlsA, which deaminates Gln into Glu, feeding the GDAR system and producing NH_3_, which captures a proton and is converted into NH_4_^+^. HdeA: chaperone protein; IM/OM: Inner and outer membrane of bacterium; The intracellular protons (H^+^) consumed during the acid stress response are contained in yellow circles; Enzymatic reactions are identified by solid arrows. Created with BioRender.com.

To survive a drastic and sudden drop in extracellular pH to values ≤2.5 without pre-adaptation, bacteria have developed different molecular strategies referred to in the literature as acid resistance (AR) mechanisms [123]. In neutrophilic Gram-negative bacteria, these AR mechanisms are most often based on the efflux of H^+^ ions, their capture during biochemical reactions, the protection of structural and functional integrity of proteins via chaperone proteins (e.g., GroE, DnaK, HdeA), and the modification of the composition of the lipid membrane [123]. In the following sections and in Figure 5, we will detail and illustrate AR mechanisms commonly present in the genus *Brucella* and those specific to novel species.

#### 4.1.1. The Urease System

Several orally acquired bacteria (*H. pylori*, *Klebsiella pneumoniae*, *Yersinia enterocolitica*, *Bacillus subtilis*), most *Brucella* species (except for *B. ovis*), and opportunistic species of the former genus *Ochrobactrum*, i.e., *O. anthropi* and *O. intermedium* [24], show urease activity. In these bacteria, urease is a nickel-containing multi-subunit enzyme that catalyzes the hydrolysis of urea to CO_2_ and ammonia (NH_3_; Figure 5). The subsequent protonation of ammonia into free ammonium (NH_4_^+^) increases pH and allows the survival of these bacteria in extremely acidic environments, especially in the stomach of mammalian hosts [124].

In classical *Brucella* species, the urease system is encoded by two large operons located on chromosome 1. Only *ureABC* of the *ure1* operon encodes the three structural subunits of the urease enzyme. Assembly and folding of the urease and capture and incorporation of the nickel ion into the active site are carried out by four accessory proteins, which are encoded by the *ureEFGD* genes and expressed in both operons. The uptake of urea is performed by different membrane transporters or channels and by an acid-activated urea transporter (UreT) located only in the ure2 operon [125]. In the classical species *B. abortus*, *B. melitensis*, and *B. suis*, the urease system is considered as a virulence trait, since it confers AR in the presence of urea under in vitro conditions at pH ≤ 2.5, and is essential for the establishment of infection in orally inoculated mice [126].

*Brucella* can be transmitted between animals and to humans by passage across the intestinal, sexual, respiratory, and conjunctival mucosae or by skin abrasions. It is not proven, however, that primary invasion after ingestion of bacteria occurs through the intestine [127]. For classical brucellae, the tonsils have been suggested as an alternative site of entry, followed by dissemination through the bloodstream. This may explain why certain urease-negative *Brucella* strains (including the species *B. ovis* and *B. abortus* bv. 1 strain 544) remain virulent for their hosts [125].

Due to the urease-positive character of the novel *Brucella* species and strains [5], we assume that the urease system may also contribute to their survival under extremely acidic conditions such as the passage through the host’s stomach following oral ingestion.

#### 4.1.2. The Amino Acid-Dependent Acid Resistance Systems

Other extreme acid stress resistance systems developed by many foodborne Gram-negative bacteria transiting through the gastrointestinal tract are based on enzymes that decarboxylate amino acids, such as arginine, glutamate, lysine, and ornithine, or deaminate glutamine. During these reactions, the protons entering the bacterial cytoplasm are sequestered in catabolites and then exported into the extracellular medium. In several bacteria, the respective activities of these systems overlap, thereby covering a fairly wide pH range, from pH 6 to <2 [123,128].

Of the 20 proteinogenic amino acids added individually to modified Gerhardt’s Minimal Medium at pH 2.5, only L-glutamate (Glu) and L-glutamine (Gln) were found to confer protection from extreme acid stress to *B. microti* [129]. Under the same experimental conditions, however, survival of the classical species *B. suis* could not be observed. Genome comparison between *B. microti* and the classical species *B. suis* reveals the existence of genes potentially involved in Glu metabolism, but intact only in *B. microti*. These genes, located at the same chromosomal locus, code for the glutamate-dependent acid resistance (GDAR) system, for a glutaminase (GlsA) producing Glu and ammonium from Gln, and for the periplasmic chaperone protein HdeA, known to play a role in the resistance of *B. abortus* to intermediate acid stress (pH 4.5) [29,130].

The GDAR system is known as the most effective AR system in *E. coli*, where it was first and extensively described [131,132], and also in other foodborne bacteria such as *Listeria monocytogenes*, *Shigella flexneri*, and *Lactococcus lactis*. In these bacteria, the GDAR system plays a decisive protective role following a drastic and sudden decrease in pH measured during the ripening process in food and in digestive juices of the host [133,134]. This system consists of: (1) one or more cytoplasmic isoenzymes with glutamate decarboxylase (GAD) activity (GadA/GadB), consuming one H^+^ ion and converting a molecule of Glu into gamma-aminobutyrate (GABA); (2) a membrane antiporter protein (GadC) which imports Glu and exports GABA (Figure 5) [131,135]. Thus, by consuming intracellular protons and exporting positive charges via GABA, this system prevents a drastic drop in intracellular pH [134]. We were able to demonstrate that the GadB and GadC proteins of *B. microti* decarboxylate Glu, resulting in the production of a GABA molecule, and export the latter into the culture medium [136]. The GDAR system enables *B. microti* to survive in a strongly acidified medium at a pH ≤ 2.5 in the presence of Glu, and it also plays a decisive role in the establishment of an infection after inoculation of mice by the oral route. The functional homology of the GDAR systems of *B. microti* and *E. coli* has also been proven by reciprocal complementation of mutant strains lacking their own GDAR system, thus allowing the restoration of bacterial survival at pH 2.5 in the presence of Glu [136]. Based on the putative GadB and GadC protein sequences, extreme acid survival assays, and a genetic complementation approach in a heterologous system of an *E. coli* mutant strain lacking its own GDAR system, we showed that in addition to *B. microti*, *B. inopinata*, and *Brucella* strains isolated from frogs and Australian rodents, as well as the marine species *B. ceti* and *B. pinnipedialis*, but not the classical terrestrial *Brucella* species, produce an active GDAR system [137]. In latter species, *gadB* and/or *gadC* are in fact inactivated by stop codons and/or frameshift mutations.

More recently, the same species and strains have been shown to possess a functional glutaminase-dependent acid resistance system (AR2-Q). This system consists of the GlnA enzyme, which is active at acidic pH, and of the GadC antiporter, allowing the import of Gln and the export of either Glu or GABA, which can be measured by qualitative colorimetric and quantitative high-performance liquid chromatography assays [128]. In addition to proton consumption and ammonia production by deamination of a molecule of Gln, glutaminase GlnA supplies Glu to the GDAR system (Figure 5) [129].

The co-occurrence of the GDAR and AR2-Q systems in these more ancestral and environmental strains [15] may explain their adaptability to extremely acidic environments, including those encountered during their passage through the gastrointestinal tract of the host [138]. In all *Brucella* species, the three genes *gadB*, *gadC*, and *glsA*, encoding the GDAR and AR2-Q system, together with the *hdeA* gene, coding for a membrane protein chaperone involved in the protection of protein structures at acidic pH, are expressed as an operon [129].

No putative proteins were annotated as GadB, GadC, and GlsA in the genomes of *B. vulpis* and *B. papionis*. In contrast, three species of the former genus *Ochrobactrum*, namely *Ochrobactrum gallinifaecis* (isolated from chicken feces), *Ochrobactrum pituitosa*, and *Ochrobactrum thiophenivorans* (isolated from the environment), encode two putative proteins, GadB and GadC, of comparable length to that of *B. microti* and with a sequence identity of at least 57 and 33%, respectively. *O. gallinifaecis* and *O. pituitosa* also encode a putative glutaminase of 309 amino acids showing at least 71% identity with GlsA of *B. microti*. The functionality of these proteins and their role in the constitution of functional AR2-Q systems remains to be proven experimentally.

Based on the currently known systems of extreme AR in *Brucella*, the genus can be divided into three groups of strains harboring (I) all three functional systems, AR2-Q, GDAR, and urease, comprising classical species isolated from marine mammals and novel and atypical strains including *B. microti, B. inopinata*, and *Brucella* isolates from frogs and Australian rodents; (II) functional urease only, comprising the classical terrestrial species; (III) none of the three AR systems, such as *B. ovis*, and *B. abortus* bv. 1 strain 544.

What may explain the distribution of AR systems within the genus Brucella? Our hypothesis is, that from a common ancestor of Brucella expressing active urease, GDAR, and glutaminase systems, the marine species and novel strains retained these AR mechanisms allowing their adaptation to a large variety of habitats. These strains are closely related to soil bacteria and therefore better adapted to natural environments with harsh conditions, where at least one of these AR systems could be a major survival benefit, depending on the availability of corresponding substrates (i.e., urea, Glu, or Gln). The phylogenetically more recent and terrestrial zoonotic Brucella species have retained only a functional urease system. Possibly, these strains do not enter their host by the intestinal mucosa, but through the genital mucous membranes or the tonsils, for example, and/or they can counter the acidity of the gastrointestinal tract exclusively by degrading the urea available in the stomach [127]. Finally, in a small group of strains such as B. ovis and B. abortus bv. 1 strain 544, no known AR mechanisms are present, which might force them to exclusively use tonsils, sexual, and vertical routes of transmission between animals. If our hypothesis is correct, this could also explain why B. ovis is not at the origin of a foodborne zoonosis.

### 4.2. Responses to Intermediate Acid Stress

*Brucella* spp. are phenotypically distinct in terms of host preference and environmental adaptability, despite their highly conserved genomic sequences, suggesting the existence of species-specific gene sequences and regulatory mechanisms. In order to identify and characterize the genetic determinants of resistance to intermediate acidity of classical *B. suis* bv. 1 strain 1330 and atypical *B. microti* strain CCM4915, a first global comparative transcriptome analysis based on RNA-Seq was recently performed at pH 4.5 and 7.0, two conditions mimicking those the bacteria face during host cell infection [30]. Confirming that large fluctuations in pH result in important variations of transcription profiles to protect the bacteria from deleterious consequences, more than 1000 genes were found to be differentially expressed under these experimental conditions. The study revealed a set of “core genes” commonly regulated in both species, including the higher expression at pH 4.5 of genes encoding the F1F0-ATPase, cytochrome oxidases, NADH-quinone oxidoreductase, the main histidine metabolic pathways, and the urease system [30]. At low pH, the more acid-sensitive species *B. suis* showed increased expression of a specifically regulated set of genes encoding, amongst others, nucleic acid-, peptidoglycan-, and LPS synthesis-related proteins, as well as diverse heat shock proteins, indicating the set-up of defense and repair mechanisms essential for structural integrity of the bacteria [30]. In contrast, the species-specific acid stress response of *B. microti* involves a significantly higher number of genes, and is characterized by the strong activation of genes participating in iron and sulfur metabolism, and of genes encoding all steps of the denitrification pathway. Furthermore, expression of the genes encoding RNA polymerase sigma factors sigma 24 (*rpoE*) and sigma 32 (*rpoH2*) is increased in *B. microti* at pH 4.5 [30]. This strengthens the hypothesis of a species-specific response to acid stress signals in *Brucella*. Interestingly, a two-component response regulator-encoding gene, also showing specifically increased expression in *B. microti* at pH 4.5, and *rpoE* are both central elements of the general stress response in Alphaproteobacteria, and have been described in *B. abortus* as being essential for in vitro stress survival and chronic murine infection [139]. Moreover, *rpoE* has been reported to be expressed consecutively to envelope damage detected by sensing of misfolded Omps and is involved in maintaining the integrity of periplasmic and outer membrane components [140]. Remarkably, two factors were deemed to be directly responsible for the increased long-term acid resistance of *B. microti* at intermediate pH: the cold shock protein CspA, which is a pseudogene in *B. suis*, and the nucleoid-associated stress protein Dps (DNA-binding protein from starved cells). Altogether, 15 acid stress-induced and potentially functional genes in *B. microti* were identified as pseudogenes in *B. suis*, adding evidence to the classification of *B. microti* as a predominantly environmental species recurrently exposed to low pH, whereas the functionality of these genes was lost during host adaptation of *B. suis*.

These results confirm that the marked phenotypical divergence between classical *B. suis* and novel *B. microti* regarding resistance to intermediate acid stress is due to point mutations and to differential gene expression. The current state of knowledge on the atypical *Brucella* spp. contributes to a better understanding of species-specific phenotypes within the genus and provides further insights into the adaptation of *Brucella* to acidic environments.

## 5. In Cellulo and In Vivo Models of *Brucella* Infection

### 5.1. General Aspects of Infection Models

As facultative intracellular pathogens, *Brucella* species have the capacity to enter and to replicate within host cells. The growing interest in the study of host–pathogen interactions over the past decades has resulted in the development of various in vitro infection models for *Brucella* spp., with a focus on professional and non-professional phagocytic cells. This approach was based on the consideration that, as air- and foodborne pathogens, *Brucella* spp. enter the host mainly via the respiratory and digestive tracts. Most frequently used cell types have been macrophages and epithelial cells mainly of human, murine, or bovine origin, but other cell types such as dendritic cells, M cells, and trophoblasts have also been studied. *Brucella* species recognized as zoonotic have been replicated in cellulo in both professional and non-professional phagocytic cells, forming distinct vacuoles with individual bacteria. These results tallied with the fact that, outside the laboratory, proliferation of these species is possible only when associated with mammalian hosts, where the pathogens can be found intracellularly within specific target organs. In vivo models of infection were set up in parallel, including laboratory animals such as mice, guinea pigs, and monkeys, as well as bovine, caprine, and pregnant sheep models [141,142,143,144]. The murine model of infection is the most commonly used animal model for *Brucella* virulence and host immune response studies. Despite the fact that mice do not show typical symptoms of brucellosis, brucellae replicate in the spleen and liver, and granulomas were described for *B. abortus* during chronic infection [145,146]. Another in vivo model is bovine jejuno-ileal Peyer’s patches, allowing the study of initial molecular and morphological interactions at this pathogen’s site of entry into the host [142].

In summary, a large panel of now well-established in cellulo and in vivo models of *Brucella* infection has been developed during the past decades to study host–pathogen interactions, allowing scientists to choose the most appropriate model for their investigations.

### 5.2. B. microti and B. inopinata BO1 in Macrophages and Murine Models of Infection

With the isolation of novel species not only from mammals but also from amphibians, fish, and environmental samples, various studies were kicked off on their capabilities to replicate intracellularly and to infect mammals using established in cellulo and in vivo models, respectively. In *Brucella*, these features correlate with pathogenicity. Intracellular behavior of atypical species was studied first with *B. microti*, isolated originally from a rodent, the common vole [15]. In the well-established models of human macrophages and human and murine macrophage-like cells, this atypical species showed increased replication when compared with classical *Brucella* species such as *B. suis* [18]. This correlated with a higher in vitro resistance of *B. microti* to acid pH, encountered in the *Brucella*-containing vacuole during the early stage of infection, and its higher replication rate in broth. Remarkably, the study of *B. microti* in the Balb/c murine model of infection with a standard dose of 10^5^ bacteria injected intraperitoneally turned out to be fatal for more than 80% of the infected animals within seven days. In CD1 and C57BL/6 mice, a 10-fold increase in the infective dose yielded similar results, suggesting that these mice were more resistant to *B. microti* than Balb/c [18]. This was the first report of a lethal outcome of murine infection by *Brucella* sp. With sublethal doses of 10^3^ and 10^4^ injected bacteria, however, infections were rapidly cleared, as compared to the long-term survival observed for *B. suis*, and these doses also conferred protection to mice against a normally lethal challenge of 10^5^ bacteria [18]. At the respective peaks of infection (four days earlier for *B. microti* than for *B. suis*), the number of bacteria in the blood was higher in *B. microti*-infected Balb/c mice, which could be a consequence of the faster growth rate of the atypical species and one possible explanation for murine death.

Another hypothesis for murine death could be the trigger of an LPS-induced septic shock. As the LPS of the classical species is of low endotoxic activity [147], this implies the existence of a structurally different LPS molecule. Interestingly, a rough mutant of *B. microti* devoid of the O-polysaccharide (O-PS) is able to replicate within macrophages, in contrast to a mutant of *B. suis* affected in the homologous gene, but which lacks the capacity to establish an acute phase of infection in the murine model [148]. In addition, the lethal infective dose in mice is four logs higher for the O-PS mutant of *B. microti* than for the wild-type strain [148], providing evidence that intact LPS is essential for this phenotype and giving credibility to the hypothesis of the presence of a structurally different LPS.

One of the major virulence factors of brucellae, the VirB type IV secretion system (T4SS; [149]), is conserved in *B. microti* [29]. Its expression is essential for replication in macrophages and murine models of infection, and its inactivation results in strong attenuation. Remarkably, the T4SS is also indispensable for lethality in mice [150]. The endpoint of a lethal outcome only a few days after infection with wild-type *B. microti* facilitates the experimental design of this infection model by shortening the observation period and making the enumeration of intrasplenic bacteria unnecessary. This will be of great importance in future identification of *B. microti* virulence factors in vivo.

*Brucella* spp. are considered as pathogens with air- and foodborne modes of transmission in human infections [151]. Besides the well-established protocol of intra-peritoneal injection, outcome of oral infection by *B. microti* was therefore examined in Balb/c mice. As a major difference, oral infection with a dose of 10^9^ bacteria was not lethal. *B. microti* could be detected and enumerated in the spleen and liver of infected animals both at five and seven days post-infection, indicating that the pathogen survives long enough in the acidic environment of the stomach to translocate across the epithelial barrier and to reach the major target organs [136].

The characteristic rapid clearing of *B. microti* infection in mice at sublethal doses has been addressed in an experimental approach, allowing researchers to study the role of the innate and adaptive host immune response and its different actors, using immunodeficient mice that lacked various cell types. Infection is controlled by B and T cells, indicating that humoral and cellular responses are involved. It is noteworthy that NK cell activity is critical for survival of mice in the absence of B and T cells, demonstrating for the first time its importance in experimental brucellosis [152]. In addition, microgranuloma similar to those described for *B. melitensis* were observed in mice during *B. microti* infection, and their early formation appears to be crucial for elimination of the pathogen [152].

Despite the fact that the common vole is a major food source for many predators and is geographically widespread through Europe, clinical and veterinary cases of infection have not yet been reported. Hence, the risk of transmission of *B. microti* from wildlife to domestic animals or humans is probably low, independent of its behavior in infection models.

**Figure 6:**
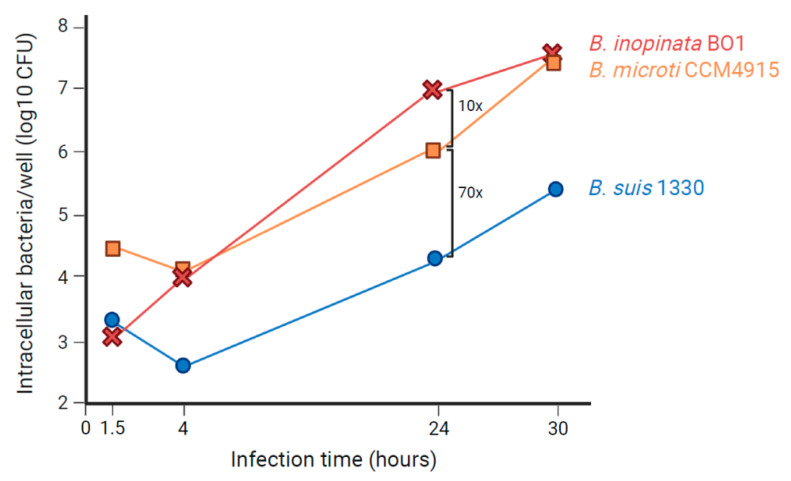
Intracellular replication profiles of *B. inopinata* BO1 and *B. microti* CCM4915 in J774 murine macrophage-like cells. Profiles of the novel species are shown in comparison to the intracellular growth curve of the classical species *B. suis* bv. 1 strain 1330. Fold-change differences in the number of viable intracellular bacteria at 24 h are indicated. Modified from [14]. Created with BioRender.com.

Later, the atypical species *B. inopinata* BO1 and *Brucella* sp. strain 83-210 from wild Australian rodents were studied in cellular and murine models of infection [88]. In contrast to *B. microti*, *B. inopinata* BO1 was isolated from a human patient [26]. In isolated macrophages, *B. inopinata* BO1 behaved as *B. microti*, showing an increased rate of intracellular replication as compared to *B. suis*, whereas strain 83–210 behaved as the classical species. Both have the capacity to kill mice, hence, sharing this characteristic phenotype with *B. microti*. At a sublethal dose, their in vivo growth profiles are, however, similar to those of the classical *B. suis*, with the set-up of a long-term infection [88]. A comparison of intramacrophagic growth profiles of *B. microti* and *B. inopinata* BO1 versus *B. suis* bv.1 strain 1330 in murine J774 macrophage-like cells is shown in Figure 6 (modified from [14]).

### 5.3. Brucella Frog Isolates in Established Infection Models

Among the recognized species of atypical brucellae, only *B. microti* and *B. inopinata* BO1 have been studied to date in experimental models of infection. Together with *Brucella* sp. strain 83-210 from rodents, they were isolated from mammalian hosts. In the meantime, an increasing number of atypical *Brucella* strains have been isolated from amphibians, and the infectious behavior of some of them has been characterized over the past five years in in cellulo and in vivo models of infection. The fate of the amphibian *Brucella* strains in mammalian cells and in the murine infection model is of particular interest since African bullfrogs have been described as the first cold-blooded host of *Brucella* [33]. It was indeed unknown, how brucellae of non-mammalian origin would behave in mammalian systems. One strain studied in these models, belonging to the “BO-clade” of strains closely related to *B. inopinata*, originated from the first reported infection of an amphibian in the U.S. and was isolated from a Pac-Man frog. Similarly to atypical species of mammalian hosts, the frog isolate revealed a significantly higher growth rate in human epithelial cells and in murine macrophages than classical *Brucella* species [36]. In addition, the number of infected cells was also higher. In parallel, 21 Gram-negative coccobacilli isolates from systemically infected and sick African bullfrogs were identified by molecular and genetic approaches as members of the genus *Brucella* and placed in a cluster with *B. inopinata* and other atypical *Brucella* species [14]. Macrophage infection experiments in murine J774 cells were performed with a selection of phenotypically diverse strains, and the number of intracellular bacteria at 24 h post-infection was 2–4 logs higher than that for the classical species *B. suis*, comparable to those obtained with *B. inopinata* BO1 and the Pac-Man frog isolate [14,36,88]. In the Balb/c murine model of infection, the strains from bullfrogs did not have the lethal effect observed with other atypical species mentioned above. Peaks of replication in the target organs spleen and liver were observed between three and seven days post-infection, comparable to other *Brucella* species, but elimination was more rapid than for *B. suis*. Despite colonization rates being different from those of *B. suis* bv. 1 strain 1330, most bullfrog strains persisted in the spleen for at least 84 days [14]. The inflammatory reaction triggered by infection with these atypical strains was apparently rather mild, because spleen and liver weights did not increase significantly. Aspects of interest for future work include investigations on a possible role of the functional flagellum in the infection of mammalian cells by the novel amphibian isolates.

### 5.4. The Chicken Embryo Model

The use of chicken embryos (CE) as an experimental in vivo model of infection has been described for *B. microti* [153]. In the past, a few publications were focused on CE infection with classical species, mainly *B. abortus* [154,155], but this model has never become a standardized model of infection, in contrast to the mammalian murine model. Wareth et al. reported effective replication of *B. microti* in CE, confirming its potential as an alternative model of infection, being easy to handle, cheaper, and with reduced ethical constraints. The *B. microti*-infected CE were histopathologically characterized by the appearance of multiple necroses in various organs such as liver, spleen, lung, and kidneys, and by death of all embryos between the second and fourth day post-inoculation [153].

**Figure 7:**
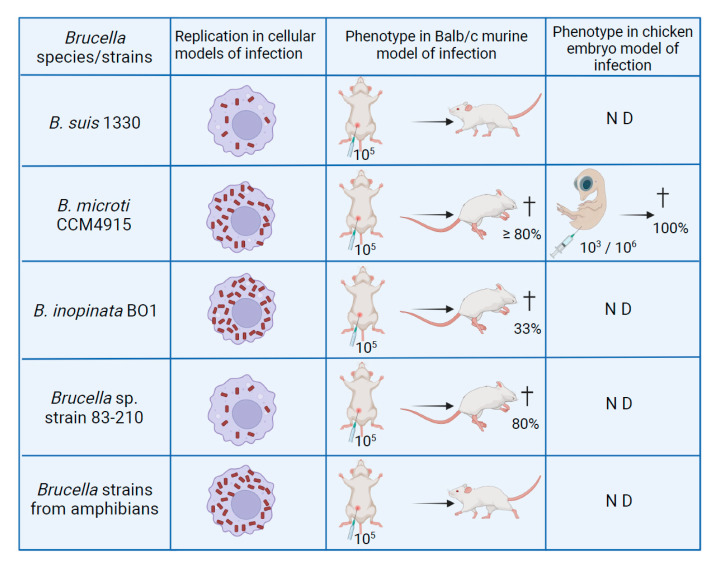
Novel *Brucella* spp. and strains in established infection models. Overview of the behavior of novel *Brucella* species and strains in cellulo and in the Balb/c murine model of infection, in comparison to the classical human pathogen *B. suis* bv. 1 strain 1330. Among the selected species, only *B. microti* has been studied in the chicken embryo model. Created with BioRender.com.

To recap the major findings obtained for atypical *Brucella* species with in vitro and in vivo infection models, it has been shown that they replicate well in macrophages of mammalian origin, and this is also true for strains isolated from amphibians. Replication rates in cellular models are significantly higher than for the classical species such as *B. suis* bv. 1 strain 1330, similar to observations made under broth culture conditions. The second remarkable point is that *B. microti* and *B. inopinata* BO1 are lethal in Balb/c mice following intraperitoneal injection at the standard dose of 10^5^ bacteria. This has never been described before for any other *Brucella* species. However, lethality was not observed in the murine model of infection using the strains isolated from African bullfrogs. The phenotypes of various atypical *Brucella* species and strains in in vivo infection models are summarized in Figure 7.

In the future, it will be of interest to characterize the behavior of the yet poorly studied novel species *B. papionis* and *B. vulpis* in in cellulo and in vivo infection models. At present, little is known about the interactions with mammalian hosts. Recently, however, as described for the major zoonotic species, *B. papionis*, associated with stillbirth in primates, proved to infect human trophoblasts, replicating preferentially in cytotrophoblasts and affecting trophoblast functions [156]. Further work will be necessary to extend these studies to other models of infection, comparing the fate of both species with that of classical and other novel *Brucella* strains.

## 6. Conclusions Demand a Harmonized Classification of *Brucella*

In the past approximately 15 years, a considerable number of additional species and strains of the genus *Brucella* have been described and at least partially characterized based on their genome sequences and on biological properties such as metabolism, acid stress resistance, and their behavior in in cellulo and in vivo models of infection.

Not surprisingly, some of the new species could be assigned to the hitherto called “classical” phylogenetic clade presenting with high genetic homology. Others, however, are genetically more diverse and diverged early, phylogenetically therefore often named “atypical”. To harmonize the nomenclature and better illustrate the relationships between “new” and “old” *Brucella*, we propose to turn away from the general designation of classical *Brucella* spp. and, based on phylogenetic analyses, rather apply the term of ***core*** *Brucella* spp., currently comprising *B. abortus*, *B. melitensis*, *B. suis, B. canis*, *B. ovis*, *B. neotomae*, *B. ceti*, and *B. pinnipedialis*, as well as the more recently described species *B. papionis* and *B. microti* [10]. Those species which do not cluster with the core should be named ***non-core*** *Brucella* spp.

This core/non-core classification of *Brucella* spp. should then be combined with a second level of classification based on the biological features of these species or strains Although genetically located within the core brucellae, *B. microti* shows atypical phenotypic traits such as increased growth rates, a broader metabolic activity, extreme acid stress resistance, and a lethal phenotype in mice, which allows its allocation to the (pheno)***atypical*** brucellae. Other strains, phylogenetically classified as non-core, e.g., various frog isolates and *B. inopinata*, are characterized by the use of ectoine or rhamnose as substrates. Furthermore, *B. inopinata* strain BO1 and Australian rodent strains, but not the exotic frog isolates, can be lethal in mice. All these novel members of the genus can therefore be affiliated to ***atypical*** brucellae as well, based on their phenotypes. Unfortunately, biological specificities have not yet been well-studied in the novel core clade species *B. papionis* and in the non-core species *B. vulpis*, which currently makes their classification as either ***typical*** or ***atypical*** *Brucella* spp. difficult.

In our opinion, the term ***atypical*** should be used only for strains and species that reveal specific biological attributes which differ from those of the other members of the genus and might be relevant for virulence, pathogenicity, host specificity, or survival in the natural environment. In the homologous group of *Brucella* spp., these biological characteristics, linked to gain or loss of function, may be a result of gene regulation depending on external triggers such as starvation or acidic stress, or of gene inactivation. For historical reasons, we propose to define the core species *B. melitensis* and *B. abortus* and their biovars as (pheno)typical references. Such contrasting juxtaposition emphasizes the variance of identified biological attributes, although we are aware that this additional classification still remains an arbitrary and artificial scheme. As a third criterion for classification, we believe that the differentiation of *Brucella* spp. based on their relevance within the One Health concept remains a valid and meaningful approach. Especially when including former *Ochrobactrum* spp. into the genus *Brucella*, this concept makes sense and has practical implications. Remarkably, the *Brucella* isolates from rodents and amphibians share various physiological characteristics, especially an expanded metabolic activity, with the former *Ochrobactrum* species, suggesting that they are better adapted to survive and proliferate in the environment than the core zoonotic *Brucella* species, and/or that they can possibly thrive in a broad range of hosts. Host jumping may therefore represent a primary mechanism of spreading for epizootic species. This phenomenon is exemplified by *B. microti* isolated from rodents, red foxes, wild boars, and soil, clearly indicating that the biological properties of these bacteria allow their positioning between merely environmental and the more host-specific zoonotic species. Adaptation of *B. microti* to intermediate acid stress, involving 15 genes that are pseudogenes in *B. suis* bv. 1 strain 1330, illustrates that genome reduction is part of the host-adaptation process of *Brucella* spp. In this way, *Brucella* species may be subdivided into ***environmental*** bacteria, exclusively ***epizootic*,** and ***zoonotic*** pathogens. In such a framework, the recognized zoonotic *Brucella* spp., which show the least heterogeneity within the genus and are hitherto named classical brucellae, may act as reference for novel, biologically diverging species, for which no clear evidence exists today that they are zoonotic. Several *B. inopinata*-like strains have been isolated from human cases, and further investigation concerning this matter may indeed confirm the zoonotic potential of novel strains.

To wrap up our proposal for harmonization of the terminology to be used for the classification of the members of the genus *Brucella*, each species or strain should be assessed according to its assignment to a phylogenetic clade (core versus non-core), its phenotypic traits (typical, such as *B. melitensis* and *B. abortus*, or atypical), and its One Health impact (zoonotic, epizootic, environmental). Many combinations appear to be possible, although it is evident that our present state of knowledge does not allow unambiguous classification of all described strains in all three criteria simultaneously.

There is no doubt that, along with increasing comprehensive understanding of the rapidly expanding genus *Brucella*, future adaptations of the recommended classifications will become necessary. However, at the moment, it is indispensable and urgent to apply the same wording for the same facts to avoid confusion among experts and stakeholders in the field.

## Data Availability

The publicly available datasets analyzed in this study can be found here: https://www.ncbi.nlm.nih.gov/datasets/genomes/?taxon=234&utm_source=assembly (accessed last on 18 January 2022).

## References

[1] Al Dahouk S., Neubauer H., Hensel A., Schöneberg I., Nöckler K., Alpers K., Merzenich H., Stark K., Jansen A. (2007). Changing epidemiology of human brucellosis, Germany, 1962–2005. Emerg. Infect. Dis..

[2] Traxler R.M., Lehman M.W., Bosserman E.A., Guerra M.A., Smith T.L. (2013). A literature review of laboratory-acquired brucellosis. J. Clin. Microbiol..

[3] Pappas G., Papadimitriou P., Akritidis N., Christou L., Tsianos E.V. (2006). The new global map of human brucellosis. Lancet Infect. Dis..

[4] Hördt A., Lopez M.G., Meier-Kolthoff J.P., Schleuning M., Weinhold L.M., Tindall B.J., Gronow S., Kyrpides N.C., Woyke T., Göker M. (2020). Analysis of 1000+ Type-Strain Genomes Substantially Improves Taxonomic Classification of Alphaproteobacteria. Front. Microbiol..

[5] Jaÿ M., Girault G., Perrot L., Taunay B., Vuilmet T., Rossignol F., Pitel P.H., Picard E., Ponsart C., Mick V. (2018). Phenotypic and Molecular Characterization of *Brucella microti*-Like Bacteria from a Domestic Marsh Frog (*Pelophylax ridibundus*). Front. Vet. Sci..

[6] Santos R.L., Souza T.D., Mol J.P.S., Eckstein C., Paixao T.A. (2021). Canine Brucellosis: An Update. Front. Vet. Sci..

[7] Suarez-Esquivel M., Ruiz-Villalobos N., Jiménez-Rojas C., Barquero-Calvo E., Chacon-Diaz C., Viquez-Ruiz E., Rojas-Campos N., Baker K.S., Oviedo-Sanchez G., Amuy E. (2017). *Brucella* neotomae Infection in Humans, Costa Rica. Emerg. Infect. Dis..

[8] Foster G., Osterman B.S., Godfroid J., Jacques I., Cloeckaert A. (2007). *Brucella ceti* sp. nov. and *Brucella pinnipedialis* sp. nov. for *Brucella* strains with cetaceans and seals as their preferred hosts. Int. J. Syst. Evol. Microbiol..

[9] Whatmore A.M., Dawson C.E., Groussaud P., Koylass M.S., King A.C., Shankster S.J., Sohn A.H., Probert W.S., McDonald W.L. (2008). Marine mammal *Brucella* genotype associated with zoonotic infection. Emerg. Infect. Dis..

[10] Whatmore A.M., Foster J.T. (2021). Emerging diversity and ongoing expansion of the genus *Brucella*. Infect. Genet. Evol..

[11] Alton G.G., Jones L.M., Angus R.D., Verger J.M. (1988). Techniques for the Brucellosis Laboratory.

[12] Wattam A.R., Inzana T.J., Williams K.P., Mane S.P., Shukla M., Almeida N.F., Dickerman A.W., Mason S., Moriyon I., O’Callaghan D. (2012). Comparative genomics of early-diverging *Brucella* strains reveals a novel lipopolysaccharide biosynthesis pathway. mBio.

[13] Zygmunt M.S., Jacques I., Bernardet N., Cloeckaert A. (2012). Lipopolysaccharide heterogeneity in the atypical group of novel emerging *Brucella* species. Clin. Vaccine Immunol..

[14] Al Dahouk S., Köhler S., Occhialini A., Jimenez de Bagues M.P., Hammerl J.A., Eisenberg T., Vergnaud G., Cloeckaert A., Zygmunt M.S., Whatmore A.M. (2017). *Brucella* spp. of amphibians comprise genomically diverse motile strains competent for replication in macrophages and survival in mammalian hosts. Sci. Rep..

[15] Scholz H.C., Hubalek Z., Sedlacek I., Vergnaud G., Tomaso H., Al Dahouk S., Melzer F., Kampfer P., Neubauer H., Cloeckaert A. (2008). *Brucella microti* sp. nov., isolated from the common vole Microtus arvalis. Int. J. Syst. Evol. Microbiol..

[16] Hofer E., Revilla-Fernandez S., Al Dahouk S., Riehm J.M., Nöckler K., Zygmunt M.S., Cloeckaert A., Tomaso H., Scholz H.C. (2012). A potential novel *Brucella* species isolated from mandibular lymph nodes of red foxes in Austria. Vet. Microbiol..

[17] Whatmore A.M., Davison N., Cloeckaert A., Al Dahouk S., Zygmunt M.S., Brew S.D., Perrett L.L., Koylass M.S., Vergnaud G., Quance C. (2014). *Brucella papionis* sp. nov., isolated from baboons (*Papio* spp.). Int. J. Syst. Evol. Microbiol..

[18] de Bagues M.P.J., Ouahrani-Bettache S., Quintana J.F., Mitjana O., Hanna N., Bessoles S., Sanchez F., Scholz H.C., Lafont V., Köhler S. (2010). The new species *Brucella* microti replicates in macrophages and causes death in murine models of infection. J. Infect. Dis..

[19] Al Dahouk S., Hofer E., Tomaso H., Vergnaud G., Le Flèche P., Cloeckaert A., Koylass M.S., Whatmore A.M., Nöckler K., Scholz H.C. (2012). Intraspecies biodiversity of the genetically homologous species *Brucella* microti. Appl. Environ. Microbiol..

[20] Hubalek Z., Scholz H.C., Sedlacek I., Melzer F., Sanogo Y.O., Nesvadbova J. (2007). Brucellosis of the common vole (*Microtus arvalis*). Vector Borne Zoonotic Dis..

[21] Scholz H.C., Nöckler K., Göllner C., Bahn P., Vergnaud G., Tomaso H., Al Dahouk S., Kämpfer P., Cloeckaert A., Maquart M. (2010). *Brucella inopinata* sp. nov., isolated from a breast implant infection. Int. J. Syst. Evol. Microbiol..

[22] Ronai Z., Kreizinger Z., Dan A., Drees K., Foster J.T., Banyai K., Marton S., Szeredi L., Janosi S., Gyuranecz M. (2015). First isolation and characterization of *Brucella* microti from wild boar. BMC Vet. Res..

[23] Mühldorfer K., Wibbelt G., Szentiks C.A., Fischer D., Scholz H.C., Zschock M., Eisenberg T. (2017). The role of ‘atypical’ *Brucella* in amphibians: Are we facing novel emerging pathogens?. J. Appl. Microbiol..

[24] Ryan M.P., Pembroke J.T. (2020). The Genus Ochrobactrum as Major Opportunistic Pathogens. Microorganisms.

[25] Scholz H.C., Hofer E., Vergnaud G., Le Flèche P., Whatmore A.M., Al Dahouk S., Pfeffer M., Krüger M., Cloeckaert A., Tomaso H. (2009). Isolation of *Brucella microti* from mandibular lymph nodes of red foxes, *Vulpes vulpes*, in lower Austria. Vector Borne Zoonotic Dis..

[26] De B.K., Stauffer L., Koylass M.S., Sharp S.E., Gee J.E., Helsel L.O., Steigerwalt A.G., Vega R., Clark T.A., Daneshvar M.I. (2008). Novel *Brucella* strain (BO1) associated with a prosthetic breast implant infection. J. Clin. Microbiol..

[27] Scholz H.C., Hubalek Z., Nesvadbova J., Tomaso H., Vergnaud G., Le Flèche P., Whatmore A.M., Al Dahouk S., Krüger M., Lodri C. (2008). Isolation of *Brucella* microti from soil. Emerg. Infect. Dis..

[28] Whatmore A.M. (2009). Current understanding of the genetic diversity of *Brucella*, an expanding genus of zoonotic pathogens. Infect. Genet. Evol..

[29] Audic S., Lescot M., Claverie J.M., Scholz H.C. (2009). *Brucella* microti: The genome sequence of an emerging pathogen. BMC Genomics.

[30] de la Garza-Garcia J.A., Ouahrani-Bettache S., Lyonnais S., Ornelas-Eusebio E., Freddi L., Al Dahouk S., Occhialini A., Köhler S. (2021). Comparative Genome-Wide Transcriptome Analysis of *Brucella* suis and *Brucella* microti Under Acid Stress at pH 4.5: Cold Shock Protein CspA and Dps Are Associated with Acid Resistance of B. microti. Front. Microbiol..

[31] Tiller R.V., Gee J.E., Lonsway D.R., Gribble S., Bell S.C., Jennison A.V., Bates J., Coulter C., Hoffmaster A.R., De B.K. (2010). Identification of an unusual *Brucella* strain (BO2) from a lung biopsy in a 52 year-old patient with chronic destructive pneumonia. BMC Microbiol..

[32] Scholz H.C., Revilla-Fernandez S., Dahouk S.A., Hammerl J.A., Zygmunt M.S., Cloeckaert A., Koylass M., Whatmore A.M., Blom J., Vergnaud G. (2016). *Brucella vulpis* sp. nov., isolated from mandibular lymph nodes of red foxes (*Vulpes vulpes*). Int. J. Syst. Evol. Microbiol..

[33] Eisenberg T., Hamann H.P., Kaim U., Schlez K., Seeger H., Schauerte N., Melzer F., Tomaso H., Scholz H.C., Koylass M.S. (2012). Isolation of potentially novel *Brucella* spp. from frogs. Appl. Environ. Microbiol..

[34] Fischer D., Lorenz N., Heuser W., Kämpfer P., Scholz H.C., Lierz M. (2012). Abscesses associated with a *Brucella* inopinata-like bacterium in a big-eyed tree frog (*Leptopelis vermiculatus*). J. Zoo Wildl. Med..

[35] Whatmore A.M., Dale E.J., Stubberfield E., Muchowski J., Koylass M., Dawson C., Gopaul K.K., Perrett L.L., Jones M., Lawrie A. (2015). Isolation of *Brucella* from a White’s tree frog (*Litoria caerulea*). JMM Case Rep..

[36] Soler-Llorens P.F., Quance C.R., Lawhon S.D., Stuber T.P., Edwards J.F., Ficht T.A., Robbe-Austerman S., O’Callaghan D., Keriel A. (2016). A *Brucella* spp. Isolate from a Pac-Man Frog (*Ceratophrys ornata*) Reveals Characteristics Departing from Classical Brucellae. Front. Cell Infect. Microbiol..

[37] Kimura M., Une Y., Suzuki M., Park E.S., Imaoka K., Morikawa S. (2017). Isolation of *Brucella* inopinata-Like Bacteria from White’s and Denny’s Tree Frogs. Vector Borne Zoonotic Dis..

[38] Latheef S., Keyburn A., Broz I., Bagnara A., Bayley C., Frith S., Dobson E.C. (2020). Atypical *Brucella* sp in captive Australian green tree frogs (*Litoria caerulea*): Clinical features, pathology, culture and molecular characterization. Aust Vet. J..

[39] Scholz H.C., Mühldorfer K., Shilton C., Benedict S., Whatmore A.M., Blom J., Eisenberg T. (2016). The Change of a Medically Important Genus: Worldwide Occurrence of Genetically Diverse Novel *Brucella* Species in Exotic Frogs. PLoS ONE.

[40] Helmick K.E., Garner M.M., Rhyan J., Bradway D. (2018). Clinicopathologic Features of Infection with Novel *Brucella* Organisms in Captive Waxy Tree Frogs (*Phyllomedusa sauvagii*) and Colorado River Toads (*Incilius alvarius*). J. Zoo Wildl. Med..

[41] Glabman R.A., Thompson K.A., Mani R., Colburn R., Agnew D.W. (2021). Atypical *Brucella* inopinata-Like Species in 2 Marine Toads. Emerg. Infect. Dis..

[42] Eisenberg T., Schlez K., Fawzy A., Volker I., Hechinger S., Curic M., Schauerte N., Geiger C., Blom J., Scholz H.C. (2020). Expanding the host range: Infection of a reptilian host (*Furcifer pardalis*) by an atypical *Brucella* strain. Antonie Van Leeuwenhoek.

[43] Eisenberg T., Risse K., Schauerte N., Geiger C., Blom J., Scholz H.C. (2017). Isolation of a novel ‘atypical’ *Brucella* strain from a bluespotted ribbontail ray (*Taeniura lymma*). Antonie Van Leeuwenhoek.

[44] Rouzic N., Desmier L., Cariou M.E., Gay E., Foster J.T., Williamson C.H.D., Schmitt F., Le Henaff M., Le Coz A., Lorleac’h A. (2021). First Case of Brucellosis Caused by an Amphibian-type *Brucella*. Clin. Infect. Dis..

[45] Jaÿ M., Freddi L., Mick V., Durand B., Girault G., Perrot L., Taunay B., Vuilmet T., Azam D., Ponsart C. (2020). *Brucella* microti-like prevalence in French farms producing frogs. Transbound. Emerg. Dis..

[46] Rajendhran J. (2021). Genomic insights into *Brucella*. Infect. Genet. Evol..

[47] Halling S.M., Tatum F.M., Bricker B.J. (1993). Sequence and characterization of an insertion sequence, IS711, from *Brucella* ovis. Gene.

[48] Ouahrani S., Michaux S., Sri Widada J., Bourg G., Tournebize R., Ramuz M., Liautard J.P. (1993). Identification and sequence analysis of IS6501, an insertion sequence in *Brucella* spp.: Relationship between genomic structure and the number of IS6501 copies. J. Gen. Microbiol..

[49] Bricker B.J., Ewalt D.R., MacMillan A.P., Foster G., Brew S. (2000). Molecular characterization of *Brucella* strains isolated from marine mammals. J. Clin. Microbiol..

[50] Dawson C.E., Stubberfield E.J., Perrett L.L., King A.C., Whatmore A.M., Bashiruddin J.B., Stack J.A., Macmillan A.P. (2008). Phenotypic and molecular characterisation of *Brucella* isolates from marine mammals. BMC Microbiol..

[51] Bricker B.J., Halling S.M. (1994). Differentiation of *Brucella* abortus bv. 1, 2, and 4, *Brucella* melitensis, *Brucella* ovis, and *Brucella* suis bv. 1 by PCR. J. Clin. Microbiol..

[52] Garcia-Yoldi D., Marin C.M., de Miguel M.J., Munoz P.M., Vizmanos J.L., Lopez-Goni I. (2006). Multiplex PCR assay for the identification and differentiation of all *Brucella* species and the vaccine strains *Brucella* abortus S19 and RB51 and *Brucella* melitensis Rev1. Clin. Chem..

[53] Wayne L.G., Brenner D.J., Colwell R.R., Grimont P.A.D., Kandler O., Krichevsky M.I., Moore L.H., Moore W.E.C., Murray R.G.E., Stackebrandt E. (1987). Report of the Ad-Hoc-Committee on Reconciliation of Approaches to Bacterial Systematics. Int. J. Syst. Bacteriol..

[54] Verger J.M., Grimont F., Grimont P.A.D., Grayon M. (1985). *Brucella*, a Monospecific Genus as Shown by Deoxyribonucleic-Acid Hybridization. Int. J. Syst. Bacteriol..

[55] Richter M., Rossello-Mora R. (2009). Shifting the genomic gold standard for the prokaryotic species definition. Proc. Natl. Acad. Sci. USA.

[56] Gee J.E., De B.K., Levett P.N., Whitney A.M., Novak R.T., Popovic T. (2004). Use of 16S rRNA gene sequencing for rapid confirmatory identification of *Brucella* isolates. J. Clin. Microbiol..

[57] Zheludkov M.M., Tsirelson L.E. (2010). Reservoirs of *Brucella* infection in nature. Biol. Bull..

[58] Mayer-Scholl A., Draeger A., Göllner C., Scholz H.C., Nöckler K. (2010). Advancement of a multiplex PCR for the differentiation of all currently described *Brucella* species. J. Microbiol. Methods.

[59] Paquet J.Y., Diaz M.A., Genevrois S., Grayon M., Verger J.M., de Bolle X., Lakey J.H., Letesson J.J., Cloeckaert A. (2001). Molecular, antigenic, and functional analyses of Omp2b porin size variants of *Brucella* spp. J. Bacteriol..

[60] Cook I., Campbell R.W., Barrow G. (1966). Brucellosis in North Queensland rodents. Aust Vet. J..

[61] Tiller R.V., Gee J.E., Frace M.A., Taylor T.K., Setubal J.C., Hoffmaster A.R., De B.K. (2010). Characterization of novel *Brucella* strains originating from wild native rodent species in North Queensland, Australia. Appl. Environ. Microbiol..

[62] Rodionova I.A., Li X., Thiel V., Stolyar S., Stanton K., Fredrickson J.K., Bryant D.A., Osterman A.L., Best A.A., Rodionov D.A. (2013). Comparative genomics and functional analysis of rhamnose catabolic pathways and regulons in bacteria. Front. Microbiol..

[63] Kim J.A., Sha Z., Mayfield J.E. (2000). Regulation of *Brucella* abortus catalase. Infect. Immun..

[64] Steele K.H., Baumgartner J.E., Valderas M.W., Roop R.M. (2010). Comparative study of the roles of AhpC and KatE as respiratory antioxidants in *Brucella* abortus 2308. J. Bacteriol..

[65] Mironov A., Seregina T., Nagornykh M., Luhachack L.G., Korolkova N., Lopes L.E., Kotova V., Zavilgelsky G., Shakulov R., Shatalin K. (2017). Mechanism of H2S-mediated protection against oxidative stress in *Escherichia coli*. Proc. Natl. Acad. Sci. USA.

[66] Pal V.K., Bandyopadhyay P., Singh A. (2018). Hydrogen sulfide in physiology and pathogenesis of bacteria and viruses. IUBMB Life.

[67] Varesio L.M., Fiebig A., Crosson S. (2021). *Brucella* ovis Cysteine Biosynthesis Contributes to Peroxide Stress Survival and Fitness in the Intracellular Niche. Infect. Immun..

[68] Carbonero F., Benefiel A.C., Alizadeh-Ghamsari A.H., Gaskins H.R. (2012). Microbial pathways in colonic sulfur metabolism and links with health and disease. Front. Physiol.

[69] Szabo C. (2018). A timeline of hydrogen sulfide (H2S) research: From environmental toxin to biological mediator. BioChem. Pharmacol.

[70] Walsh B.J.C., Giedroc D.P. (2020). H2S and reactive sulfur signaling at the host-bacterial pathogen interface. J. Biol. Chem..

[71] Vazquez-Torres A., Baumler A.J. (2016). Nitrate, nitrite and nitric oxide reductases: From the last universal common ancestor to modern bacterial pathogens. Curr. Opin. Microbiol..

[72] Al Dahouk S., Scholz H.C., Tomaso H., Bahn P., Göllner C., Karges W., Appel B., Hensel A., Neubauer H., Nöckler K. (2010). Differential phenotyping of *Brucella* species using a newly developed semi-automated metabolic system. BMC Microbiol..

[73] Meyer M.E., Cameron H.S. (1961). Metabolic characterization of the genus *Brucella*. II. Oxidative metabolic patterns of the described biotypes. J. Bacteriol..

[74] Das P., Lahiri A., Lahiri A., Chakravortty D. (2010). Modulation of the arginase pathway in the context of microbial pathogenesis: A metabolic enzyme moonlighting as an immune modulator. PLoS Pathog..

[75] Haine V., Dozot M., Dornand J., Letesson J.J., De Bolle X. (2006). NnrA is required for full virulence and regulates several *Brucella* melitensis denitrification genes. J. Bacteriol..

[76] Loisel-Meyer S., Jimenez de Bagues M.P., Basseres E., Dornand J., Kohler S., Liautard J.P., Jubier-Maurin V. (2006). Requirement of norD for *Brucella* suis virulence in a murine model of in vitro and in vivo infection. Infect. Immun..

[77] Philippot L. (2005). Denitrification in pathogenic bacteria: For better or worst?. Trends Microbiol..

[78] Baek S.H., Rajashekara G., Splitter G.A., Shapleigh J.P. (2004). Denitrification genes regulate *Brucella* virulence in mice. J. Bacteriol..

[79] Breitschwerdt E.B., Kordick D.L. (2000). Bartonella infection in animals: Carriership, reservoir potential, pathogenicity, and zoonotic potential for human infection. Clin. Microbiol. Rev..

[80] Ronneau S., Moussa S., Barbier T., Conde-Alvarez R., Zuniga-Ripa A., Moriyon I., Letesson J.J. (2016). *Brucella*, nitrogen and virulence. Crit Rev. Microbiol..

[81] Cameron H.S., Holm L.W., Meyer M. (1952). Comparative metabolic studies on the genus *Brucella*. I. Evidence of a urea cycle from glutamic acid metabolism. J. Bacteriol..

[82] Doern G.V. (2000). Detection of selected fastidious bacteria. Clin. Infect. Dis..

[83] Gerhardt P. (1958). The nutrition of Brucellae. Bacteriol. Rev..

[84] Plommet M. (1991). Minimal requirements for growth of *Brucella* suis and other *Brucella* species. Zent. Bakteriol..

[85] Zuniga-Ripa A., Barbier T., Lazaro-Anton L., de Miguel M.J., Conde-Alvarez R., Munoz P.M., Letesson J.J., Iriarte M., Moriyon I. (2018). The Fast-Growing *Brucella suis* Biovar 5 Depends on Phosphoenolpyruvate Carboxykinase and Pyruvate Phosphate Dikinase but Not on Fbp and GlpX Fructose-1,6-Bisphosphatases or Isocitrate Lyase for Full Virulence in Laboratory Models. Front. Microbiol..

[86] Gopalsamy S.N., Ramakrishnan A., Shariff M.M., Gabel J., Brennan S., Drenzek C., Farley M.M., Gaynes R.P., Cartwright E.J. (2021). Brucellosis Initially Misidentified as Ochrobactrum anthropi Bacteremia: A Case Report and Review of the Literature. Open Forum Infect. Dis..

[87] Corbel M.J., Brinley Morgan W.J. (1982). Classification of the genus *Brucella*: The current position. Rev. Sci. Tech..

[88] Jimenez de Bagues M.P., Iturralde M., Arias M.A., Pardo J., Cloeckaert A., Zygmunt M.S. (2014). The new strains *Brucella* inopinata BO1 and *Brucella* species 83-210 behave biologically like classic infectious *Brucella* species and cause death in murine models of infection. J. Infect. Dis..

[89] Suarez-Esquivel M., Chaves-Olarte E., Moreno E., Guzman-Verri C. (2020). *Brucella* Genomics: Macro and Micro Evolution. Int. J. Mol. Sci..

[90] Colin R., Ni B., Laganenka L., Sourjik V. (2021). Multiple functions of flagellar motility and chemotaxis in bacterial physiology. FEMS Microbiol. Rev..

[91] O’Callaghan D., Whatmore A.M. (2011). *Brucella* genomics as we enter the multi-genome era. Brief. Funct. Genom..

[92] Moreno E., Cloeckaert A., Moriyon I. (2002). *Brucella* evolution and taxonomy. Vet. Microbiol..

[93] Chain P.S., Comerci D.J., Tolmasky M.E., Larimer F.W., Malfatti S.A., Vergez L.M., Aguero F., Land M.L., Ugalde R.A., Garcia E. (2005). Whole-genome analyses of speciation events in pathogenic Brucellae. Infect. Immun..

[94] Rajashekara G., Glasner J.D., Glover D.A., Splitter G.A. (2004). Comparative whole-genome hybridization reveals genomic islands in *Brucella* species. J. Bacteriol..

[95] Tsolis R.M., Seshadri R., Santos R.L., Sangari F.J., Lobo J.M., de Jong M.F., Ren Q., Myers G., Brinkac L.M., Nelson W.C. (2009). Genome degradation in *Brucella* ovis corresponds with narrowing of its host range and tissue tropism. PLoS ONE.

[96] Wattam A.R., Foster J.T., Mane S.P., Beckstrom-Sternberg S.M., Beckstrom-Sternberg J.M., Dickerman A.W., Keim P., Pearson T., Shukla M., Ward D.V. (2014). Comparative phylogenomics and evolution of the Brucellae reveal a path to virulence. J. Bacteriol..

[97] Wattam A.R., Williams K.P., Snyder E.E., Almeida N.F., Shukla M., Dickerman A.W., Crasta O.R., Kenyon R., Lu J., Shallom J.M. (2009). Analysis of ten *Brucella* genomes reveals evidence for horizontal gene transfer despite a preferred intracellular lifestyle. J. Bacteriol..

[98] Zhong Z., Wang Y., Xu J., Chen Y., Ke Y., Zhou X., Yuan X., Zhou D., Yang Y., Yang R. (2012). Parallel gene loss and acquisition among strains of different *Brucella* species and biovars. J. Microbiol..

[99] Sheppard S.K., Didelot X., Meric G., Torralbo A., Jolley K.A., Kelly D.J., Bentley S.D., Maiden M.C., Parkhill J., Falush D. (2013). Genome-wide association study identifies vitamin B5 biosynthesis as a host specificity factor in Campylobacter. Proc. Natl. Acad. Sci. USA.

[100] Barbier T., Zuniga-Ripa A., Moussa S., Plovier H., Sternon J.F., Lazaro-Anton L., Conde-Alvarez R., De Bolle X., Iriarte M., Moriyon I. (2018). *Brucella* central carbon metabolism: An update. Crit. Rev. Microbiol..

[101] Barbier T., Collard F., Zuniga-Ripa A., Moriyon I., Godard T., Becker J., Wittmann C., Van Schaftingen E., Letesson J.J. (2014). Erythritol feeds the pentose phosphate pathway via three new isomerases leading to D-erythrose-4-phosphate in *Brucella*. Proc. Natl. Acad. Sci. USA.

[102] Essenberg R.C., Seshadri R., Nelson K., Paulsen I. (2002). Sugar metabolism by Brucellae. Vet. Microbiol..

[103] Spaans S.K., Weusthuis R.A., van der Oost J., Kengen S.W. (2015). NADPH-generating systems in bacteria and archaea. Front. Microbiol..

[104] Rytter H., Jamet A., Ziveri J., Ramond E., Coureuil M., Lagouge-Roussey P., Euphrasie D., Tros F., Goudin N., Chhuon C. (2021). The pentose phosphate pathway constitutes a major metabolic hub in pathogenic Francisella. PLoS Pathog..

[105] Marais A., Mendz G.L., Hazell S.L., Megraud F. (1999). Metabolism and genetics of Helicobacter pylori: The genome era. Microbiol. Mol. Biol. Rev..

[106] Hofreuter D. (2014). Defining the metabolic requirements for the growth and colonization capacity of Campylobacter jejuni. Front. Cell Infect. Microbiol..

[107] Tettelin H., Saunders N.J., Heidelberg J., Jeffries A.C., Nelson K.E., Eisen J.A., Ketchum K.A., Hood D.W., Peden J.F., Dodson R.J. (2000). Complete genome sequence of Neisseria meningitidis serogroup B strain MC58. Science.

[108] Canback B., Andersson S.G., Kurland C.G. (2002). The global phylogeny of glycolytic enzymes. Proc. Natl. Acad. Sci. USA.

[109] Seo J.S., Chong H., Park H.S., Yoon K.O., Jung C., Kim J.J., Hong J.H., Kim H., Kim J.H., Kil J.I. (2005). The genome sequence of the ethanologenic bacterium Zymomonas mobilis ZM4. Nat. Biotechnol..

[110] Barbier T., Nicolas C., Letesson J.J. (2011). *Brucella* adaptation and survival at the crossroad of metabolism and virulence. FEBS Lett..

[111] Brissac T., Ziveri J., Ramond E., Tros F., Kock S., Dupuis M., Brillet M., Barel M., Peyriga L., Cahoreau E. (2015). Gluconeogenesis, an essential metabolic pathway for pathogenic Francisella. Mol. Microbiol..

[112] Blume M., Nitzsche R., Sternberg U., Gerlic M., Masters S.L., Gupta N., McConville M.J. (2015). A Toxoplasma gondii Gluconeogenic Enzyme Contributes to Robust Central Carbon Metabolism and Is Essential for Replication and Virulence. Cell Host Microbe..

[113] Machelart A., Willemart K., Zuniga-Ripa A., Godard T., Plovier H., Wittmann C., Moriyon I., De Bolle X., Van Schaftingen E., Letesson J.J. (2020). Convergent evolution of zoonotic *Brucella* species toward the selective use of the pentose phosphate pathway. Proc. Natl. Acad. Sci. USA.

[114] Lazaro-Anton L., de Miguel M.J., Barbier T., Conde-Alvarez R., Munoz P.M., Letesson J.J., Iriarte M., Moriyon I., Zuniga-Ripa A. (2020). Glucose Oxidation to Pyruvate Is Not Essential for *Brucella* suis Biovar 5 Virulence in the Mouse Model. Front. Microbiol..

[115] Meyer M.E., Morgan W.J. (1962). Metabolic characterization of *Brucella* strains that show conflicting identity by biochemical and serological methods. Bull. World Health Organ..

[116] Varesio L.M., Willett J.W., Fiebig A., Crosson S. (2019). A Carbonic Anhydrase Pseudogene Sensitizes Select *Brucella* Lineages to Low CO_2_ Tension. J. Bacteriol..

[117] Finan T.M., Weidner S., Wong K., Buhrmester J., Chain P., Vorholter F.J., Hernandez-Lucas I., Becker A., Cowie A., Gouzy J. (2001). The complete sequence of the 1683-kb pSymB megaplasmid from the N2-fixing endosymbiont *Sinorhizobium meliloti*. Proc. Natl. Acad. Sci. USA.

[118] Hermann L., Mais C.N., Czech L., Smits S.H.J., Bange G., Bremer E. (2020). The ups and downs of ectoine: Structural enzymology of a major microbial stress protectant and versatile nutrient. Biol. Chem..

[119] Mais C.N., Hermann L., Altegoer F., Seubert A., Richter A.A., Wernersbach I., Czech L., Bremer E., Bange G. (2020). Degradation of the microbial stress protectants and chemical chaperones ectoine and hydroxyectoine by a bacterial hydrolase-deacetylase complex. J. Biol. Chem..

[120] Thuoc D.V., Hien T.T., Sudesh K. (2019). Identification and characterization of ectoine-producing bacteria isolated from Can Gio mangrove soil in Vietnam. Ann. Microbiol..

[121] Decamp A., Michelo O., Rabbat C., Laroche C., Grizeau D., Pruvost J., Goncalves O. (2021). A New, Quick, and Simple Protocol to Evaluate Microalgae Polysaccharide Composition. Mar. Drugs.

[122] Flamholz A., Noor E., Bar-Even A., Liebermeister W., Milo R. (2013). Glycolytic strategy as a tradeoff between energy yield and protein cost. Proc. Natl. Acad. Sci. USA.

[123] Lund P., Tramonti A., De Biase D. (2014). Coping with low pH: Molecular strategies in neutralophilic bacteria. FEMS Microbiol. Rev..

[124] Sangari F.J., Cayon A.M., Seoane A., Garcia-Lobo J.M. (2010). *Brucella* abortus ure2 region contains an acid-activated urea transporter and a nickel transport system. BMC Microbiol..

[125] Sangari F.J., Seoane A., Rodriguez M.C., Aguero J., Garcia Lobo J.M. (2007). Characterization of the urease operon of *Brucella* abortus and assessment of its role in virulence of the bacterium. Infect. Immun..

[126] Bandara A.B., Contreras A., Contreras-Rodriguez A., Martins A.M., Dobrean V., Poff-Reichow S., Rajasekaran P., Sriranganathan N., Schurig G.G., Boyle S.M. (2007). *Brucella* suis urease encoded by ure1 but not ure2 is necessary for intestinal infection of BALB/c mice. BMC Microbiol..

[127] Gorvel J.P., Moreno E., Moriyon I. (2009). Is *Brucella* an enteric pathogen?. Nature Rev. Microbiol..

[128] Pennacchietti E., D’Alonzo C., Freddi L., Occhialini A., De Biase D. (2018). The Glutaminase-Dependent Acid Resistance System: Qualitative and Quantitative Assays and Analysis of Its Distribution in Enteric Bacteria. Front. Microbiol..

[129] Freddi L., Damiano M.A., Chaloin L., Pennacchietti E., Al Dahouk S., Köhler S., De Biase D., Occhialini A. (2017). The Glutaminase-Dependent System Confers Extreme Acid Resistance to New Species and Atypical Strains of *Brucella*. Front. Microbiol..

[130] Valderas M.W., Alcantara R.B., Baumgartner J.E., Bellaire B.H., Robertson G.T., Ng W.L., Richardson J.M., Winkler M.E., Roop R.M. (2005). Role of HdeA in acid resistance and virulence in *Brucella* abortus 2308. Vet. Microbiol..

[131] De Biase D., Tramonti A., Bossa F., Visca P. (1999). The response to stationary-phase stress conditions in *Escherichia coli*: Role and regulation of the glutamic acid decarboxylase system. Mol. Microbiol..

[132] Castanie-Cornet M.P., Penfound T.A., Smith D., Elliott J.F., Foster J.W. (1999). Control of acid resistance in *Escherichia coli*. J. Bacteriol..

[133] Cotter P.D., Gahan C.G., Hill C. (2001). A glutamate decarboxylase system protects Listeria monocytogenes in gastric fluid. Mol. Microbiol..

[134] De Biase D., Pennacchietti E. (2012). Glutamate decarboxylase-dependent acid resistance in orally acquired bacteria: Function, distribution and biomedical implications of the gadBC operon. Mol. Microbiol..

[135] De Biase D., Tramonti A., John R.A., Bossa F. (1996). Isolation, overexpression, and biochemical characterization of the two isoforms of glutamic acid decarboxylase from *Escherichia coli*. Protein Expr. Purif..

[136] Occhialini A., Jimenez de Bagues M.P., Saadeh B., Bastianelli D., Hanna N., De Biase D., Köhler S. (2012). The glutamic acid decarboxylase system of the new species *Brucella* microti contributes to its acid resistance and to oral infection of mice. J. Infect. Dis..

[137] Damiano M.A., Bastianelli D., Al Dahouk S., Köhler S., Cloeckaert A., De Biase D., Occhialini A. (2015). Glutamate decarboxylase-dependent acid resistance in *Brucella* spp.: Distribution and contribution to fitness under extremely acidic conditions. Appl. Environ. Microbiol..

[138] Grassini G., Pennacchietti E., Cappadocio F., Occhialini A., De Biase D. (2015). Biochemical and spectroscopic properties of *Brucella* microti glutamate decarboxylase, a key component of the glutamate-dependent acid resistance system. FEBS Open Bio.

[139] Kim H.S., Caswell C.C., Foreman R., Roop R.M., Crosson S. (2013). The *Brucella* abortus general stress response system regulates chronic mammalian infection and is controlled by phosphorylation and proteolysis. J. Biol. Chem..

[140] Raivio T.L., Silhavy T.J. (2001). Periplasmic stress and ECF sigma factors. Ann. Rev. Microbiol..

[141] Silva T.M., Costa E.A., Paixao T.A., Tsolis R.M., Santos R.L. (2011). Laboratory animal models for brucellosis research. J. Biomed. Biotechnol..

[142] Rossetti C.A., Drake K.L., Siddavatam P., Lawhon S.D., Nunes J.E., Gull T., Khare S., Everts R.E., Lewin H.A., Adams L.G. (2013). Systems biology analysis of *Brucella* infected Peyer’s patch reveals rapid invasion with modest transient perturbations of the host transcriptome. PLoS ONE.

[143] Elzer P.H., Hagius S.D., Davis D.S., DelVecchio V.G., Enright F.M. (2002). Characterization of the caprine model for ruminant brucellosis. Vet. Microbiol..

[144] Hensel M.E., Garcia-Gonzalez D.G., Chaki S.P., Hartwig A., Gordy P.W., Bowen R., Ficht T.A., Arenas-Gamboa A.M. (2020). Vaccine Candidate *Brucella* melitensis 16MDeltavjbR Is Safe in a Pregnant Sheep Model and Confers Protection. mSphere.

[145] Montaraz J.A., Winter A.J. (1986). Comparison of living and nonliving vaccines for *Brucella* abortus in BALB/c mice. Infect. Immun..

[146] Young E.J., Gomez C.I., Yawn D.H., Musher D.M. (1979). Comparison of *Brucella* abortus and *Brucella* melitensis infections of mice and their effect on acquired cellular resistance. Infect. Immun..

[147] Rasool O., Freer E., Moreno E., Jarstrand C. (1992). Effect of *Brucella* abortus lipopolysaccharide on oxidative metabolism and lysozyme release by human neutrophils. Infect. Immun..

[148] Ouahrani-Bettache S., Jimenez De Bagues M.P., De La Garza J., Freddi L., Bueso J.P., Lyonnais S., Al Dahouk S., De Biase D., Köhler S., Occhialini A. (2019). Lethality of *Brucella* microti in a murine model of infection depends on the wbkE gene involved in O-polysaccharide synthesis. Virulence.

[149] O’Callaghan D., Cazevieille C., Allardet-Servent A., Boschiroli M.L., Bourg G., Foulongne V., Frutos P., Kulakov Y., Ramuz M. (1999). A homologue of the Agrobacterium tumefaciens VirB and Bordetella pertussis Ptl type IV secretion systems is essential for intracellular survival of *Brucella* suis. Mol. Microbiol..

[150] Hanna N., Jimenez de Bagues M.P., Ouahrani-Bettache S., El Yakhlifi Z., Köhler S., Occhialini A. (2011). The virB operon is essential for lethality of *Brucella* microti in the Balb/c murine model of infection. J. Infect. Dis..

[151] Pappas G., Akritidis N., Bosilkovski M., Tsianos E. (2005). Brucellosis. N. Engl. J. Med..

[152] de Bagues M.P.J., de Martino A., Quintana J.F., Alcaraz A., Pardo J. (2011). Course of infection with the emergent pathogen *Brucella* microti in immunocompromised mice. Infect. Immun..

[153] Wareth G., Böttcher D., Melzer F., Shehata A.A., Roesler U., Neubauer H., Schoon H.A. (2015). Experimental infection of chicken embryos with recently described *Brucella* microti: Pathogenicity and pathological findings. Comp. Immunol. Microbiol. Infect. Dis..

[154] Detilleux P.G., Cheville N.F., Deyoe B.L. (1988). Pathogenesis of *Brucella* abortus in chicken embryos. Vet. Pathol..

[155] Gay K., Damon S.R. (1951). A yolk sac technique for the routine isolation of *Brucella*; injection of clotted blood specimens into embryonating eggs with recovery of all three species. Public Health Rep..

[156] Garcia-Mendez K.B., Hielpos S.M., Soler-Llorens P.F., Arce-Gorvel V., Hale C., Gorvel J.P., O’Callaghan D., Keriel A. (2019). Infection by *Brucella* melitensis or *Brucella* papionis modifies essential physiological functions of human trophoblasts. Cell Microbiol..

